# Human IFT-A complex structures provide molecular insights into ciliary transport

**DOI:** 10.1038/s41422-023-00778-3

**Published:** 2023-02-13

**Authors:** Meiqin Jiang, Vivek Reddy Palicharla, Darcie Miller, Sun-Hee Hwang, Hanwen Zhu, Patricia Hixson, Saikat Mukhopadhyay, Ji Sun

**Affiliations:** 1 Department of Structural Biology, St. Jude Children’s Research Hospital, Memphis, TN USA; 2 Department of Cell Biology, University of Texas Southwestern Medical Center, Dallas, TX USA

**Keywords:** Cryoelectron microscopy, Cilia

## Abstract

Intraflagellar transport (IFT) complexes, IFT-A and IFT-B, form bidirectional trains that move along the axonemal microtubules and are essential for assembling and maintaining cilia. Mutations in IFT subunits lead to numerous ciliopathies involving multiple tissues. However, how IFT complexes assemble and mediate cargo transport lacks mechanistic understanding due to missing high-resolution structural information of the holo-complexes. Here we report cryo-EM structures of human IFT-A complexes in the presence and absence of TULP3 at overall resolutions of 3.0–3.9 Å. IFT-A adopts a “lariat” shape with interconnected core and peripheral subunits linked by structurally vital zinc-binding domains. TULP3, the cargo adapter, interacts with IFT-A through its N-terminal region, and interface mutations disrupt cargo transport. We also determine the molecular impacts of disease mutations on complex formation and ciliary transport. Our work reveals IFT-A architecture, sheds light on ciliary transport and IFT train formation, and enables the rationalization of disease mutations in ciliopathies.

## Introduction

Cilia are hair-like organelles in eukaryotic cells and play fundamental roles in locomotion and stimulus sensing.^1,2^ Cilium assembly and maintenance require multi-subunit intraflagellar transport (IFT) protein complexes: IFT-A and IFT-B, which form repetitive structures known as the IFT trains.^3–7^ Powered by kinesins and dynein 2, IFT trains shuttle cargos along axonemal microtubules to maintain the steady-state ciliary length.^8–10^ IFT-A regulates retrograde transport in cilia, as lack of IFT-A subunits results in shortened cilia with the apical accumulation of tubulin, IFT-B and kinesin-II, similar to the disruption of the retrograde IFT motor dynein 2.^11–15^

IFT-A is also essential for transporting key membrane or membrane-associated proteins, such as GPCRs, ion channels and lipidated proteins, into cilia.^16–19^ Mutations of IFT-A subunits affect the trafficking of multiple lipidated proteins in *Chlamydomonas*,^20^ and lead to the mislocalization of a putative guanylyl cyclase in chemosensory cilia of *C. elegans*.^21^ The tubby family protein, TULP3, functions as an adapter linking IFT-A to cargos in mammalian cells.^16,18,22^ IFT-A and TULP3 mediate ciliary transport of the orphan GPCR, GPR161, which has been implicated in the negative regulation of vertebrate hedgehog signaling.^23–26^ In *Drosophila*, the altered localization of TRPV channels in the specialized mechanosensory chordotonal cilia,^27^ caused by IFT-A mutations, phenocopies the deficiency of the tubby homolog, dTULP.^28^

Mutations in IFT complexes lead to numerous ciliopathies involving the brain, kidney, skeleton and eyes.^29,30^ Numerous genetic mutations have been reported in IFT-A subunits in human diseases, including short-rib thoracic dysplasia (SRTD), cranioectodermal dysplasia (CED), asphyxiating thoracic dystrophy (ATD), nephronophthisis (NPHP), Senior-Loken syndromes (SLSN), retinitis pigmentosa (RP), and most recently, the autosomal dominant polycystic kidney disease spectrum.^31–36^

For its importance in human physiology and ciliopathies, much effort has been taken to structurally characterize the IFT system. Recent cryo-electron tomography (cryo-ET) work has elucidated the anterograde IFT trains and the IFT stepwise assembly process, which has largely improved our structural understanding of ciliary transport.^37,38^ In addition, integrative models of IFT-A by combining cryo-ET and chemical crosslinking mass spectrometry, and single-particle cryo-electron microscopy (cryo-EM) analysis have also been published during the preparation of our manuscript^39,40^ and have greatly enriched our current knowledge in the IFT-A assembly and train formation. However, these models do not seem to reach a consensus, likely due to the lack of a high-resolution structure of IFT-A holo-complex, hindering the mechanistic dissection of the diverse and conserved roles of IFT-A in ciliary assembly, maintenance and trafficking, as well as the rational interpretation of disease mutations. Here we report the much sought-after high-resolution structures of the human IFT-A complex as a basis for future structural-function analyses.

## Results

### Structural determination of the IFT-A complex

**Fig. 1:**
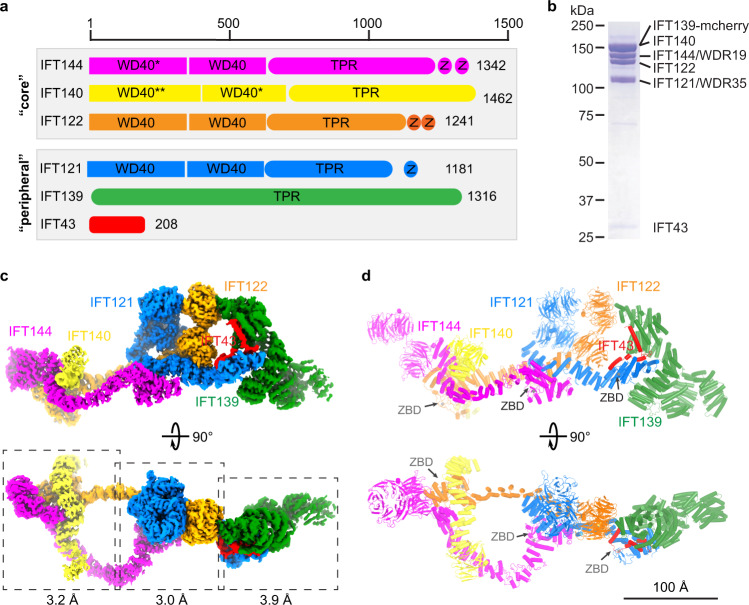
Structure of the IFT-A complex. **a** Domain architecture of the IFT-A subunits. The “core” and “peripheral” subcomplexes are indicated in gray boxes. WD40, TPR and Z stand for WD40, TPR-like and zinc-binding domains, respectively. * indicates that the domain is resolved at low resolution but we could use AF2 model to do rigid body docking. ** indicates that the domain is not resolved due to flexibility. **b** SDS-PAGE of the purified IFT-A complex. IFT139 is tagged with mCherry. **c** Cryo-EM density of the IFT-A complex. IFT144, IFT140, IFT139, IFT122, IFT121 and IFT43 are colored in magenta, yellow, green, orange, blue and red, respectively. The same color codes are used in the manuscript unless otherwise noted. The local refinement resolutions are indicated. **d** Structural model of the IFT-A complex shown as cartoons. The ZBDs are indicated by gray arrows.

For biochemical and structural characterization, we co-expressed and purified the human IFT-A complex, which contains six polypeptides: IFT144 (WDR19), IFT140, IFT139 (TTC21B/THM1), IFT122, IFT121 (WDR35) and IFT43, with IFT144–IFT140–IFT122 and IFT139–IFT121–IFT43 forming the “core” and “peripheral” subcomplexes, respectively (Fig. 1a). Purified IFT-A complex showed biochemical homogeneity on the SDS-PAGE and gel filtration chromatography (Fig. 1b; Supplementary information, Fig. S1a, b). Single-particle cryo-EM analysis was performed to obtain a final density map (Fig. 1c) composed of volumes with overall resolutions ranging from 3.0 Å to 3.9 Å (Fig. 1c; Supplementary information, Figs. S2a–d, S3a and Table S1).

The IFT-A complex has a dimension of ~380 Å × 150 Å × 140 Å and adopts a “lariat” shape (Fig. 1c, d; Supplementary information, Fig. S2c). Our cryo-EM maps allow modeling of almost full-length IFT121, IFT122, IFT139 and IFT144 (Fig. 1d). WD40 domains of IFT140 (residues 1–770) are too flexible for de novo model building and show little interaction with the rest of the IFT-A complex (Supplementary information, Fig. S3b), providing a possible explanation for the observation that truncated IFT140 without WD40 domains was able to partially rescue flagella formation in *Chlamydomonas*.^20^ The WD40-A of IFT144 (residues 1–350) also shows flexibility (Supplementary information, Fig. S3b, c). We docked AlphaFold2 (AF2)^41,42^ models of the IFT140 WD40-B and IFT144 WD40-A into the cryo-EM map with minimal refinement during model building. For IFT43, we resolved residues 121–192 (Supplementary information, Fig. S3e).

IFT121, IFT122, IFT140 and IFT144 manifest sequence and structural similarity, containing two WD40 domains followed by TPR-like domains, and resemble the architecture of β′-COP^43^ (PDB: 3MKQ; Supplementary information, Fig. S3d, e). However, TPR-like domains of IFT-A subunits are structurally unique — an insertion helix is found between TPR2 and TPR3, resulting in opposite topologies of the two neighboring TPR-like repeats (Supplementary information, Fig. S3e, f). IFT139, made of TPR or TPR-like repeats, forms a right-handed superhelix and is distally localized in the IFT-A complex (Fig. 1c, d; Supplementary information, Fig. S3b, e). This structural arrangement aligns with the observation that IFT139 is dispensable for IFT-A assembly.^19^ The resolved C-terminal half of IFT43 has four helices named HA–HD (Supplementary information, Fig. S3e).

**Fig. 2:**
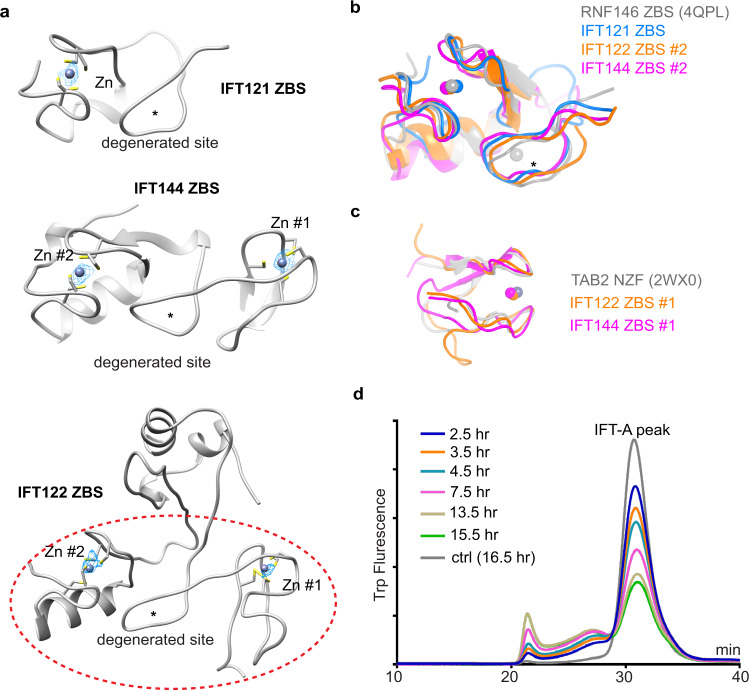
The ZBDs of IFT121, IFT122 and IFT144. **a** The structures of ZBDs of IFT121, IFT122 and IFT144. The ZBD of IFT122 is indicated by red dashed lines. The zinc densities are shown as meshes, and coordinating cysteines are shown as sticks. Stars indicate the possible degenerated zinc-binding sites. The contour level for the zinc is 30–35× RMSD. **b** Superimposition of the IFT121 ZBS, IFT122 ZBS #2 and IFT144 ZBS #2 with the ZBS of RNF146 (PDB: 4QPL) with RMSD of 0.229, 0.283 and 0.515, respectively. **c** Superimposition of the IFT122 ZBS #1 and IFT144 ZBS #1 with the Tab2 NZF (PDB: 2WX0) with RMSD of 2.090 and 1.259, respectively. **d** Fluorescence-detection size-exclusion chromatography (FSEC) traces of IFT-A complex stability with TPEN treatment by monitoring the tryptophan fluorescence.

**Fig. 3:**
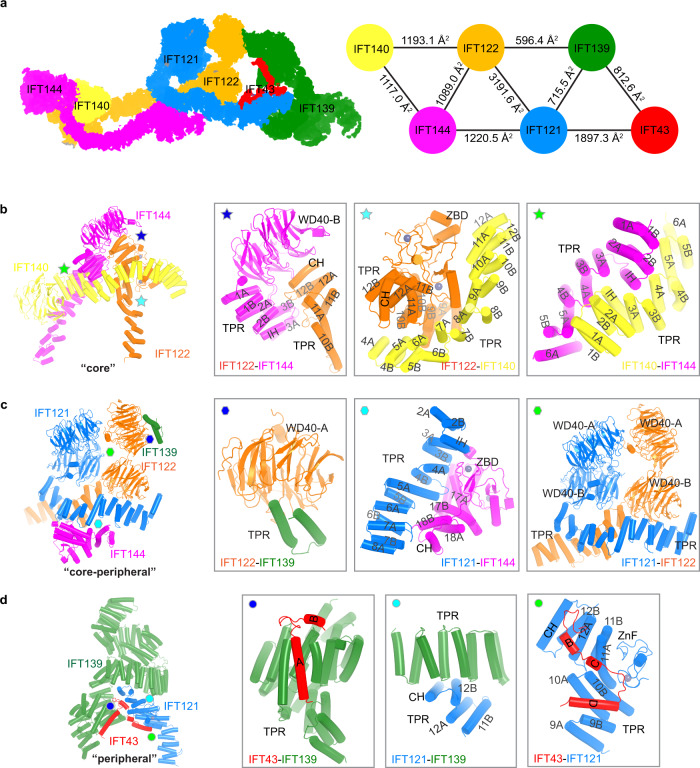
Inter-subunit interactions within IFT-A. **a** An overview of inter-subunit interactions within IFT-A complexes. Left: the interaction networks are shown as 2D projections. Right: the interaction network and interface areas are shown. **b** Interactions within the “core” subcomplex. TPR-like domain helices are labeled as in Supplementary information, Fig. S3e. **c** Interactions between the “core” and “peripheral” subcomplexes. **d** Interactions within the “peripheral” subcomplex.

The IFT-A structure revealed five unexpected zinc-binding sites (ZBSs): two in IFT144, two in IFT122 and one in IFT121, containing strong Zn^2+^ densities (Figs. 1a, d, 2a). IFT121, IFT122 and IFT144 each have an additional degenerated site (Fig. 2a). Localized after TPR-like domains (Fig. 1a), the zinc-binding domains (ZBDs) were characterized as “CXXC” motifs in previous sequence analyses.^44^ Structures of the ZBS #2 of IFT122 and IFT144 or the ZBS of IFT121 resemble the ZBS domain of RNF146, an E3 ligase (PDB: 4QPL)^45^ (Fig. 2b). ZBS #1 of both IFT122 and IFT144 are superimposable to the NZF domain of Tab2 (PDB: 2WX0)^46^ (Fig. 2c). The ZBDs found at IFT43–IFT121, IFT122–IFT140 and IFT121–IFT144 interfaces could be critical in mediating inter-subunit interactions and thus essential for IFT-A assembly and stability (Figs. 1d and 3b–d). Indeed, TPEN (an ion chelator with a high affinity to zinc ions) treatment destabilizes the complex over time (Fig. 2d). Thus, the ZBDs are critical for IFT-A stability.

### Organization of the IFT-A complex

The IFT-A structure allows us to dissect the inter-subunit interactions and characterize IFT-A assembly at atomic details (Fig. 3; Supplementary information, Fig. S3e). In the “core” subcomplex (Fig. 3b), IFT122 inserts its C-terminal part of the TRP-like domain between WD40-B and TPR1–3 of IFT144 (residues 350–730), supporting the observation that residues 357–653 of IFT144 are required for IFT122 interaction.^47^ The TPR-like domain of IFT140 (TPR4–12) wraps around ZBDs and TPR9–12 of IFT122. IFT140 and IFT144 interact through their TPR1–6 in a head-to-tail manner. Of note, TPR-like domains of IFT122 and IFT144, well-resolved and shaping two opposite sides of the “lariat” loop, structurally bridge the “core” and “peripheral” subcomplexes (Fig. 1c, d) and would be critical for the assembly of the holo-IFT-A complex as inferred previously in mammalian cells and *Chlamydomonas*.^16,44^ Therefore, the first exon deletions or early truncation alleles of these two subunits (*Ift122*^*med1Δ1–3*^, *Ift144*^*dmhd*^) by abolishing IFT-A complex formation leads to severe ciliogenesis defects and impairment of hedgehog signaling in the ventral neural tube.^48,49^ In contrast, the start site mutant of *Ift122* (*Ift122*^*sopb*^)^25^ and point mutant of *Ift144* (*Ift144*^*twt*^)^48^ exhibit relatively normal cilium morphology and expansion of ventral progenitors in the neural tube with characteristic high hedgehog signaling.

In the “peripheral” subcomplex (Fig. 3d), IFT139 is dispensable for IFT-A assembly.^19^ Indeed, we could purify stable IFT-A complexes without IFT139 (Supplementary information, Fig. S1c). Therefore, null mutant of *Ift139* (*Ttc21b*^*aln*^) has relatively normal cilium morphology compared to *Ift144* knockout (KO) mutant.^19,50^ Part of the IFT121–IFT43 complex^17^ inserts into the superhelix groove of IFT139 and uncovers direct interaction between the HA–HB helices of IFT43 and IFT139^19^ (Fig. 3d). The IFT-A peripheral subunits have also been shown to form a subcomplex,^51^ and our structural data align with this possibility.

Interactions between the “core” and “peripheral” subcomplexes are mediated by IFT122–IFT139, IFT121–IFT144 and IFT121–IFT122 interfaces with estimated buried surface areas of 596.4 Å^2^, 1220.5 Å^2^ and 3191.6 Å^2^, respectively (Fig. 3a, c). Notably, IFT139 dips its last two helices into the first WD40 domain of IFT122 (Fig. 3c). Although our structural observation is mostly consistent with the previous molecular analyses based on biochemical and genetic data,^16,19,44,47^ it shows significant differences from the recent cryo-ET-based integrative structural models (discussed below).^40,52^

### IFT-A–TULP3 complex structure and insights into cargo transport

We determined the cryo-EM structure of IFT-A in complex with TULP3 to gain insights into ciliary transport. TULP3 is an adapter protein that mediates the interaction between IFT-A and membrane-embedded or -associated cargos. We co-purified the IFT-A–TULP3 complex for cryo-EM analysis (Supplementary information, Fig. S1d) and found an extra density next to IFT140 and IFT122 which is corresponding to TULP3 (Supplementary information, Fig. S4a). To improve the resolution, we determined an overall 4.3-Å structure of the IFT-A (without IFT139)–TULP3 complex (Supplementary information, Figs. S1e and S4b–e). We excluded IFT139 to simplify structural determination, as it is not involved in TULP3 interaction (Supplementary information, Fig. S4a, b). The final resolution of the TULP3-binding region reaches an overall resolution of ~3.6 Å by focused refinement (Supplementary information, Fig. S4b), allowing confident modeling of the complex (Supplementary information, Fig. S4c–e and Table S1).

**Fig. 4:**
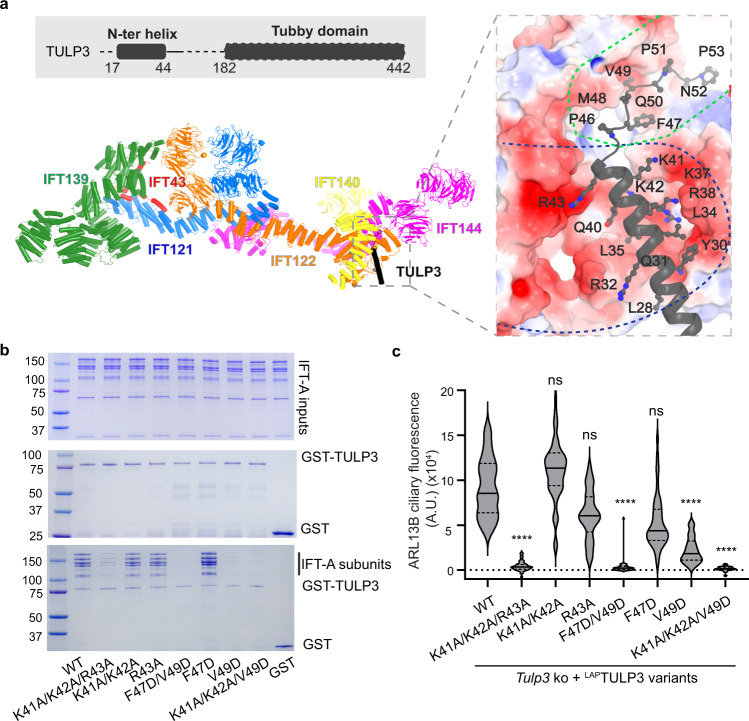
Structure of the IFT-A–TULP3 complex. **a** The TULP3 interaction interface with IFT-A. The relative position of TULP3 in an IFT-A complex and the domain scheme of TULP3 are shown with unresolved regions indicated by dash lines. TULP3 is colored black. The charged surface is indicated by blue dash lines, and the surface with mixed chemical properties by green dash lines. The surface potential (red to blue: –5 kT/e to 5 kT/e) of IFT-A is calculated using the APBS plugin in PyMOL. **b** GST pull-down assays between IFT-A complexes and GST-tagged TULP3 WT and mutants. Top: IFT-A input. Middle: GST and GST-TULP3 input. Bottom: Pull-down beads. **c** *Tulp3* KO IMCD3 line stably expressing LAP-tagged WT TULP3 or the indicated mutants were grown to confluency, serum starved for 36 h before fixation and immunostained for ARL13B, GFP, acetylated tubulin and counterstained for DNA. ARL13B ciliary fluorescence intensities were plotted as a violin plot (images in Supplementary information, Fig. S6). *n* = 50 cilia per condition. ****P < 0.0001; ns not significant with respect to WT.

TULP3 interacts with the TPR-like domain of IFT140 and the TPR-like and zinc finger domains of IFT122 via its N-terminal region (Fig. 4a; Supplementary information, Fig. S5a–c). TULP3 is a bipartite molecule with the N-terminal part mediating IFT-A interactions and the C-terminal tubby domain mediating cargo recognition and phosphoinositide 4,5-bisphosphate interactions.^16,18,53^ We resolved the N-terminal region of TULP3 encompassing residues 19–54 with a helix (residues 19–44) and a loop (residues 45–54); the tubby domain is not resolved due to flexibility (Supplementary information, Fig. S5a). The electrostatic surface representation showed that the helix-docking surface is negatively charged, and the loop-binding interface has mixed chemical properties with both hydrophobic and hydrophilic residues (Fig. 4a; Supplementary information, Fig. S5a–c).

To test how IFT-A–TULP3 interactions affect cargo transportation, we mutated the TULP3 interface residues at both the helical and loop regions and examined cargo trafficking in the mutants. By stably expressing wild-type (WT) and helical/loop mutants of TULP3 in a *Tulp3* KO IMCD3 cell line,^22^ we monitored the ciliary levels of ARL13B (a palmitoylated cargo) and GPR161, whose ciliary transportation is dependent on TULP3.^18,22,23,54,55^ We determined that mutations in the TULP3 helical region (K41A/K42A/R43A), loop region (F47D/V49D) or a combination of both (K41A/K42A/V49D) could significantly disrupt TULP3–IFT-A interaction and reduce ARL13B and GPR161 trafficking to cilia (Fig. 4b, c; Supplementary information, Figs. S6, S7). These results validated the critical role of the resolved interface in cargo trafficking.

**Fig. 5:**
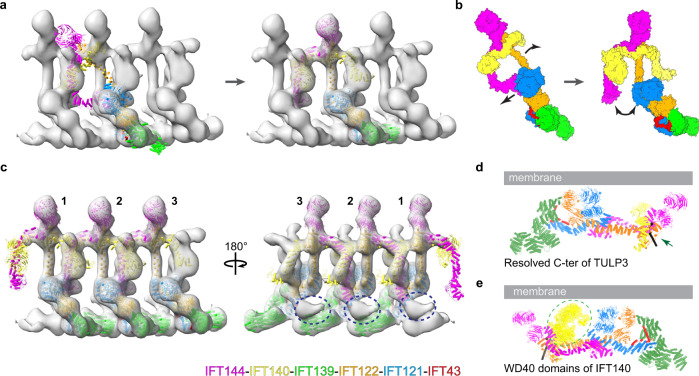
Docking of IFT-A into cryo-ET maps. **a** Left: rigid body-based docking of the IFT-A model to the cryo-ET map (EMDB-26791, *Chlamydomonas reinhardtii*). The train was generated by docking 3 EMDB-26791 into EMDB-4304, followed by the UCSF Chimera vop maximum command to combine the 3 EMDB-26791 maps. Right: docking of the IFT-A model to the cryo-ET map by treating IFT-A as 2 rigid bodies, followed by refinement of the docking through molecular dynamics simulation. **b** The conformational change between IFT-A models in **a**. The black arrows indicate conformational changes. **c** A model of the IFT-A chain. Blue dashed circles indicate unexplained density. **d** The orientation of TULP3 terminus. The resolved C-terminus of TULP3 is indicated by a green arrow. **e** The position of the WD40 domains of IFT140. The WD40 domains are shown as yellow surfaces and indicated by a dashed circle.

We then performed molecular docking of the human IFT-A structure into available in-situ cryo-ET maps (EMDB-4304, EMDB-15259, EMDB-26791)^37,38,40^ of *Chlamydomonas* IFT trains to understand IFT-A train assembly and IFT-A orientation during cargo transport. Rigid body docking failed to yield perfect fitting (Fig. 5a; Supplementary information, Fig. S10a), which was achievable if we divided IFT-A into two halves: IFT139–IFT43–IFT121–IFT122 (1–770) and IFT144–IFT140–IFT122 (770–end) (Fig. 5a, c). This docking result requires large conformational changes, including the bending of the IFT122 TPR domain and the rupture of the interface between IFT144 (TPR17–18 and ZBD) and IFT121 (TPR3–7) (Fig. 5b). Such observation leads us to ask what could potentially cause the difference between our cryo-EM structure and the cryo-ET model (Fig. 5b). It is possible that conformational changes occur upon IFT-A monomer to polymer transition, which could be regulated by posttranslational modification, protein binding partner(s) or other unknown factors. Interestingly, unexplained densities exist between IFT139 (n) and IFT144 (n + 2) (Fig. 5c). The region that IFT144 uses for interaction with IFT121 in our cryo-EM structure (Fig. 3c) is next to this density. The differences may also result from species-specific differences, the low-resolution nature of cryo-ET maps or different functional states of IFT-A — for example, states during retrograde transport or a non-polymerization state. Further studies will be needed to explore these possibilities. Lastly, our docking analyses suggest that IFT-A polymerization is mainly mediated by interactions between IFT144/IFT140 and IFT140/IFT121 of the neighboring subunit (Fig. 5c; Supplementary information, Fig. S10b).

The molecular docking also revealed the membrane orientation of IFT-A complexes. As expected, the last resolved residue of TULP3, which is linked to the cargo-interacting tubby domain,^18,22^ points toward the membrane (Fig. 5d). We modeled both WD40 domains of IFT140 by combining AF2 model and the low-resolution cryo-EM map (Fig. 5e). All WD40-A domains of IFT121, IFT122, IFT140 and IFT144 face the cell membrane (Fig. 5d, e) and are accessible for protein–protein interaction except the top face of IFT122, which is occupied by IFT139 (Fig. 3c), and the side face of IFT122 WD40-A is exposed. Because WD40 domains are usually involved in protein–protein interaction, they could play a role in cargo recognition and/or membrane proximity during anterograde transport. Indeed, the IFT140 WD40 domains were proposed to be important for cargo transport,^20^ and mutations in the WD40 domains of IFT122 have been implicated in ciliary trafficking defects with minor effects in ciliogenesis.^47^

### IFT-A disease mutations

**Fig. 6:**
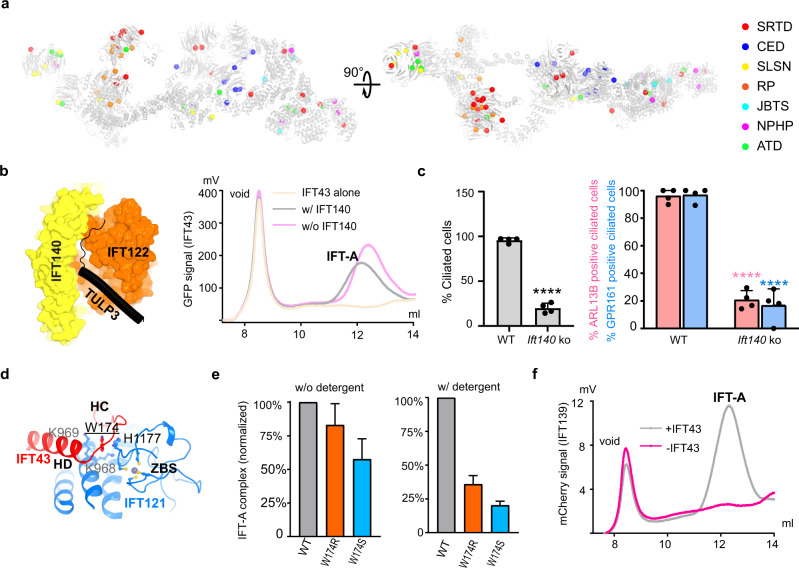
IFT-A disease mutations. **a** IFT-A disease mutations are mapped based on disease types. SRTD short-rib thoracic dysplasia, CED cranioectodermal dysplasia, SLSN Senior-Loken syndrome, RP retinitis pigmentosa, JBTS Joubert syndrome, NPHP nephronophthisis, ATD asphyxiating thoracic dystrophy. Note: for a complete mapping of the disease mutations, we modeled the WD40-A of IFT140 with the following approach: we took the AF2 model of IFT140 WD40 domains and then aligned with our resolved WD40-B to get the position of WD40-A. **b** Left: Role of IFT140 in TULP3 binding. Right: FSEC traces of IFT-A complex formation with or without IFT-140 by monitoring the GFP fluorescence of GFP-IFT43. **c** Impact of *Ift140* KO on ciliary transport. WT and *Ift140* KO MEFs were serum starved upon confluency for 48 h before fixation. Fixed cells were immunostained for ARL13B or GPR161, acetylated tubulin, γ-tubulin, and counterstained for DNA (shown in Supplementary information, Fig. S9a, b). Cilia were counted from 2 coverslips each from 2 experiments, *n* > 300/condition. Data represent mean ± SD. ****P < 0.0001 with respect to WT. **d** Interaction details surrounding Trp174 at the interface between the HC–HD region of IFT43 and the ZBS of IFT121. **e** IFT-A complex formation in the presence of GFP-tagged IFT43^W174^ mutants with or without adding detergents. IFT-A complex amount (*y*-axis) is calculated b*y* integrating the chromatography peak and normalized to WT (*n* = 3). **f** The IFT-A complex formation in the presence (gray) and absence (magenta) of GFP-IFT43 by FSEC.

We mapped sites of missense mutations onto the structure based on disease types or surface accessibility to gain molecular insights into the IFT-A-related ciliopathies (Fig. 6a; Supplementary information, Fig. S8a, b). Disease mutations distribute over the complex except for TPR-like domains of IFT122 and IFT144 that form the loop of the “lariat” (Fig. 6a). It is possible that these domains are structurally isolated and may be less involved in mediating protein–protein interaction.

The structure provides a blueprint for rationalizing IFT-A disease mutations at the molecular level. *Ift140* mutations are associated with multiple diseases. Monoallelic truncation mutations of *IFT140* have recently been shown to be associated with mild late-onset polycystic kidney disease,^35^ whereas the bi-allelic variants are associated with syndromic ciliopathies such as Mainzer-Saldino Syndrome (also known as SRTD9).^34^ With fluorescence-detection size-exclusion chromatography (FSEC),^56^ we revealed that IFT140, a core subunit, is not required for IFT-A assembly like IFT139 (Fig. 6b) and IFT-A subcomplex could be purified without IFT140 (Supplementary information, Fig. S9c). As IFT140 accounts for almost half of the TULP3-binding surface from the structure (Fig. 6b), we hypothesize that one functional consequence of *IFT140* truncation mutations is the disruption of TULP3-mediated cargo transport. Indeed, *Ift140* KO MEFs have reduced ciliary levels of TULP3 cargos: ARL13B and GPR161 in the remaining cilia (Fig. 6c; Supplementary information, Fig. S9a, b).

Furthermore, mechanistic characterization of disease mutations provides directions for therapeutic interventions. Here we use IFT43^W174R^ as a case study. The IFT43^W174R^ mutation showed reduced IFT43 protein amount in cells and defects in ciliogenesis, leading to short rib polydactyly syndrome with distinctive campomelia (SRTD18).^57^ Because Trp174 is located at the interface between the ZBS of IFT121 and the HC–HD loop of IFT43 (Fig. 6d), we postulate that IFT43^W174R^ could destabilize the IFT-A complex, which leads or adds to the reduced IFT43 protein level.^57^ To test this hypothesis, we monitored the IFT-A complex formation using FSEC.^56^ IFT43^W174R^ leads to a less stable IFT-A holo-complex, and chemical stresses like mild detergents have a more significant disruption effect on the mutant complex (Fig. 6e; Supplementary information, Fig. S9d). In addition, IFT43^W174S^ has similar results, confirming the role of IFT43^W174^ in maintaining the entirety of the IFT-A holo-complex (Fig. 6e; Supplementary information, Fig. S9d). These data support that the IFT43^W174^ mutations weaken the interaction between IFT43 and the rest IFT-A components, and therefore, pharmacological tools that could “glue” IFT43 and IFT121 hold the potential for correcting generic defects caused by IFT43^W174^ mutations in SRTD18 patients. Furthermore, stable IFT43 is required for IFT139 assembly to the IFT-A complex. IFT43 is localized between IFT139 and the rest of the IFT-A subunits (Fig. 3a). IFT139 fails to incorporate (Fig. 6f; Supplementary information, Fig. S9e) without IFT43 and largely dissociates from the complex in the presence of both IFT43^W174^ mutations and mild detergents (Supplementary information, Fig. S9f), consistent with previous genetic analyses.^58^

## Discussion

Our study reveals the molecular basis underlying IFT-A architecture, assembly and stability and bridges the gap between cross-species data on conserved functions of the complex and recent in situ cryo-ET work. Unexpectedly, we find the role of multiple structurally vital ZBDs in maintaining IFT-A complex stability. A recent study that performed IFT-A purification and reconstitution in the presence of EGTA presented a highly flexible core complex, possibly due to the disruption of ZBDs.^39^

The IFT-A structures that we describe help dissect the emerging crosstalk between IFT-A and other IFT or motor complexes and shed light on train formation and ciliary transport. Trafficking of cargos by IFT-A involves membrane-proximate interactions between the complex, adapter(s) and cargos. The IFT-A–TULP3 structure uncovers the atomic basis underlying IFT-A and TULP3 interactions essential for cargo transport into the cilium membrane. By docking the IFT-A structure into the low-resolution cryo-ET maps, we further reveal the membrane orientation of IFT-A and TULP3 pertinent to cargo transport. These insights allow us to determine the molecular impacts of disease mutations on complex formation and ciliary trafficking.

Recent work combining low-resolution cryo-ET maps and AI-based modeling methods like AF2 proposed integrative models of IFT-A complexes^37,40,52^ and provided valuable information for IFT-A assembly. However, the resulting models seem to be different from each other and from our structures (Supplementary information, Fig. S10c–e). Besides providing more precise subunit interaction information, our study shows that IFT140 has less intensive interaction with the rest of the IFT-A components in the monomer compared to the integrative model^40^ (Supplementary information, Fig. S10c). When docking IFT-A into the low-resolution cryo-ET map, we placed IFT140 and IFT144 in different regions of the cryo-ET map (Supplementary information, Fig. S10d). Additionally, our results suggest a more compact TPR domain structure of IFT144 and the possible presence of an unexplained density (Fig. 5c; Supplementary information, Fig. S10d, e) that could regulate the switch between “IFT144-attached” conformation in IFT-A monomers and “IFT144-detached” conformation in IFT-A polymers (Fig. 5b). Such observation also provides a plausible explanation for why IFT-A does not form polymers before entering cilia. We think that AI-based modeling of large IFT-A complexes in low-resolution IFT-A train maps is challenging, as it is difficult to identify a single non-repetitive unit, not to mention potential densities from other complexes like IFT-B or even unknown factors. Therefore, our high-resolution structural information is highly valuable for confidently guiding future biochemical and cell-based studies on IFT.

## Materials and methods

### Protein expression and purification

The cDNAs encoding human IFT121 (AAH36659.1), IFT122 (AAH28353.1), IFT140 (NP_055529.2), IFT139 (BC055424.1) were purchased from Horizon Discovery and cDNAs encoding human IFT43 (NP_001096034.1), IFT144 (NP_079408.3), TULP3 (NP_003315.2) were synthesized by GENEWIZ. IFT140 and IFT122 were cloned into a BacMam expression vector without any tags^59^ (construct 1). A preScission protease cleavage site followed by a GFP tag was engineered at the C-terminus of IFT43.^60^ IFT121 and IFT43 were cloned into one BacMam expression vector (construct 2). IFT144 was cloned into a BacMam expression vector without any tags (construct 3). IFT139 was cloned into a BacMam expression vector with (construct 4) or without an N-terminal mCherry tag (construct 5). TULP3 was cloned into a BacMam expression vector with an N-terminal mCherry tag (construct 6). Constructs used for IFT-A expression and purification were summarized in Supplementary information, Fig. S1a.

For expression and purification of the IFT-A complex, baculoviruses for constructs 1–4 were generated separately using the Bac-to-Bac system according to the manufacturer’s instructions (Invitrogen). A P2 virus mixture of constructs 1–4 (1:1:1:1) was used for the transfection of HEK293F cells for protein expression. Briefly, for a 600 mL culture of HEK293F cells (~2–3 × 10^6^ cells/mL) in Freestyle 293 medium (Gibco) supplemented with 2% FBS (Gibco), ~80 mL P2 virus mixture was used. Infected cells were incubated at 37 °C overnight, followed by adding 10 mM sodium butyrate to induce protein expression. Cells were cultured at 30 °C for another 48–60 h before harvesting.^59^

Cell pellets from 1.2 L culture were resuspended in 50 mL of lysis buffer (20 mM Tris-HCl, pH 8.0, 200 mM NaCl, 2 mM DTT, protease inhibitors) and then lysed by adding 17 mL of detergent mixture of lauryl maltose neopentyl glycol/cholesteryl hemisuccinate (LMNG/CHS, 8:1 mass ratio, 4% stock solubilized in 20 mM Tris-HCl, pH 8.0, 200 mM NaCl) with a 30 min incubation at 4 °C. Soluble IFT-A was separated from the insoluble fraction by high-speed centrifugation (16,500 rpm for 45 min) and incubated with 2 mL CNBR-activated Sepharose beads (GE Healthcare) coupled with 2 mg high-affinity GFP nanobodies.^61^ The GFP tag was cleaved by preScission proteases overnight at 4 °C. IFT-A was further purified by size exclusion chromatography with a Superose 6, 10/300 GL column (GE Healthcare) equilibrated with 20 mM Tris-HCl, pH 8.0, 200 mM NaCl and 2 mM DTT. Fractions of the peak around 12.5 mL were pooled and concentrated to ~6–8 mg/mL (OD_280_) using a 100-kDa MWCO centrifugal device (Amicon). The protein sample was immediately used for cryo-EM grid preparation.

The same protocol for protein expression and purification was applied to IFT-A (–IFT139) complex (constructs 1–3), IFT-A–TULP3 complex (constructs 1–3, 5, 6) and IFT-A–TULP3 (–IFT139) complex (constructs 1–3, 6).

For IFT140 disease mutation study, IFT122 was cloned into a BacMam expression vector without any tags. IFT-A (–IFT140) complex was expressed and purified using the same protocol mentioned above.

### GST pull-down assay

For GST pull-down assay, WT and mutant constructs of TULP3 were generated. Briefly, the variant genes were cloned into the modified pGEX plasmid (Sigma) with a GST tag at the N-terminus. The GST-tagged TULP3 constructs were overexpressed in BL21 (DE3) in LB media at 16 °C for 16 h after induction with 0.4 mM IPTG at an OD_600_ of 0.8. Cell pellets were resuspended in the lysis buffer (20 mM Tris-HCl, pH 8.0, 200 mM NaCl, 5% glycerol, 2 mM DTT, and 1 mM PMSF) and lysed by sonication. The lysates were spun at 15,000 rpm for 45 min. The soluble TULP3 proteins were incubated with the glutathione sepharose beads (GE Healthcare) for 2 h and washed with 50 mL lysis buffer. The bound proteins were eluted with lysis buffer containing 20 mM GSH. Further purification was performed by size exclusion chromatography (Superdex 200 Increase 10/300 GL column (GE Healthcare)) in a buffer containing 20 mM Tris-HCl, pH 8.0, 200 mM NaCl, and 2 mM DTT. The protein was concentrated using the 30-kDa MWCO centrifugal device (Amicon).

The GST pull-down assay was performed as follows. 10 µg (0.13 nmol) GST-tagged TULP3 variants were incubated with 20 µL GST beads for 1.0 h in 400 µL pull-down buffer (20 mM Tris-HCl, pH 8.0, 70 mM or 100 mM NaCl, 2 mM DTT) at 4 °C. Then, beads were washed 3 times (8 min each) with 900 µL pull-down buffer and incubated with 330 µg (0.42 nmol) IFT-A in 500 µL pull-down buffer at 4 °C for 2 h. The unbound proteins were then washed away with 900 µL pull-down buffer (3 times), and the washed beads were boiled with pull-down buffer and SDS-PAGE protein loading buffer. The pull-down results were examined by SDS-PAGE gel staining with Coomassie Blue. The GST protein was used as the negative control.

### Cryo-EM sample preparation, data collection and processing

2 mM Fluorinated Fos-choline-8 (FFC-8) were added to protein samples before grid freezing. 3.5 µL protein samples were applied to plasma-cleaned UltrAufoil R1.2/1.3 300 mesh grids or Quantifoil R1.2/1.3 300 mesh grids (EMS). After a 20-s incubation, grids were blotted for 3 s under a blot force of –3 at 16 °C and 100% humidity and plunged into liquid ethane using a Vitrobot Mark IV (FEI). The grids were loaded onto a 200 kV Talos Arctica or a 300-keV Titan Krios transmission electron microscope (FEI) with a K3 direct electron detector (Gatan) and an energy filter.

Images of IFT-A complex were recorded at super-resolution mode using EPU (Krios, 300 kV) with a super-resolution pixel size of 0.5285 Å (magnification of 81,000×) and a defocus range of –0.6 µm to –1.8 µm. Each micrograph was dose-fractionated into 60 frames with a dose rate of 1.11 e^–^/Å^2^/s and a total exposure time of 4 s. Similar conditions were applied to the image acquisition for IFT-A–TULP3 (–IFT139) complex. Datasets of IFT-A (–IFT139) complex and IFT-A–TULP3 complex were collected at super-resolution mode using SerialEM with a super-resolution pixel size of 0.413 Å (Krios, 300 kV) and 0.6572 Å (Arctica, 200 kV), respectively. Detailed image acquisition parameters of all the datasets were summarized in Supplementary information, Table S1.

Super-resolution image stacks were gain-normalized, binned by 2 with Fourier cropping, and corrected for beam-induced motion using MotionCor2.^62^ All subsequent processing was performed on motion-corrected summed images with dose weighting. Contrast transfer function parameters were estimated using Gctf or CTFFIND4.^63^ Micrographs with ctf resolution worse than 6 Å were excluded. Particles were picked and 2D classifications were carried out in cryoSPARC.^64^ The initial 3D models were generated by cryoSPARC ab initio reconstruction. Multiple rounds of heterogeneous refinement were performed to remove bad particles, followed by non-uniform refinement in cryoSPARC. Focused refinement was carried out to improve the resolution of the final reconstruction. Resolutions were calculated at FSC = 0.143. Local resolutions of density maps were estimated in cryoSPARC.

For the IFT-A complex, a composite map was generated as follows. First, sub-volume 1 (sub-vol. 1) and sub-volume 2 (sub-vol. 2) were obtained by performing local refinement on the IFT-A full complex sample. Here, sub-vol. 1 and sub-vol. 2 mainly contain IFT139 and WD40 domains of IFT121–IFT122 (Supplementary information, Fig. S2a). Similarly, sub-vol. 3 and sub-vol. 4 were obtained using the IFT-A (–IFT139) dataset (Supplementary information, Fig. S2b). Sub-vol. 3 and sub-vol. 4 mainly contain WD40s of IFT121–IFT122 and IFT144–IFT140. Then, sub-vol. 1–4 were merged to generate a composite map in UCSF Chimera. Here, we use IFT-A (–IFT139) sample to resolve sub-vol. 3 and sub-vol. 4, because IFT-A (–IFT139) is less flexible and yields higher quality maps than the full complex.

For the IFT-A–TULP3 complex, we first showed that IFT139 is not involved in TULP3 binding using a 200-keV Arctica dataset (Supplementary information, Fig. S4a and Table S1). Then a Krios dataset was collected using the IFT-A(–IFT139)–TULP3 sample. Local refinement was performed to obtain two sub-volumes: sub-vol. 1 and sub-vol. 2 (Supplementary information, Fig. S4b), which were combined in UCSF Chimera to generate a composite map of the IFT-A–TULP3 (–IFT139) complex. A full IFT-A–TULP3 complex model was generated by combining IFT-A and IFT-A(–IFT139)–TULP3 structures for illustration purposes.

### Model building and refinement

Assignment of IFT121, IFT122, IFT144 and IFT140 was guided by cryo-EM maps and biochemical data. De novo model building was performed for IFT121, IFT122, IFT144 and IFT140 in COOT.^65^ Homology models of WD40 domains were generated by the SWISS-MODEL^66^ server to guide initial model building before AlphaFold2 was available. AlphaFold2^41^ models were docked into the cryo-EM map using UCSF Chimera^67^ and UCSF ChimeraX^68^ to guide later model building. Manual rebuilding was carried out in COOT.^65^ Structural models were refined using phenix.real_space_refine^69^ with secondary structure restraints imposed. The final model was validated using the Molprobity.^70^ Figures were prepared using PyMOL (Schrödinger, LLC) and UCSF Chimera.

For docking of IFT-A model into cryo-ET maps (EMDB-4304, EMDB-15259, EMDB-26791),^37,38,40^ we performed rigid body docking using UCSF Chimera^67^ and UCSF ChimeraX.^68^ To acquire a good model of IFT-A train, the IFT-A model was split into two lobes: lobe1 contains IFT139, IFT43, IFT121 and IFT122 (1–770), and lobe2 contains IFT144, IFT122 (771–end) and IFT140. Lobe1 can fit well into cryo-ET map (EMDB-26791, 23 Å) using ChimeraX. Most of lobe2 can fit into the same map, except both the TPR domains of IFT144 and IFT140 (combine our model and AF2 model). We further split these 2 TPR domains from lobe2 and manually positioned them to best fit the map. Then we combined the separated model in one pdb file and used Namdinator^71^ to perform molecular dynamics flexible fitting (MDFF). After several rounds of alternating manual adjustment and MDFF performance, we obtained the final model that fits the cyro-ET map well. The map for IFT-A train was generated by docking of 3 EMDB-26791 into EMDB-4304, followed by the UCSF Chimera vop maximum command to combine the 3 EMDB-26791 maps.

### FSEC

For IFT43^W174R^ disease mutation analysis, IFT43, IFT43^W174R^ and IFT43^W174S^ were cloned into a BacMam expression vector with a GFP tag at the C-terminus.

HEK293F cells cultured in a 6-well dish were transfected with 2.5 μg of DNA (0.5 μg for each construct of IFT-A subunits) using Lipofectamine 3000. After incubation at 37 °C for 8–12 h, cells were supplemented with 10 mM sodium butyrate and incubated at 30 °C for another 48–72 h before harvesting. IFT-A expressing cells were washed with DPBS and resuspended in 130 µL buffer A (20 mM Tris-HCl, pH 8.0, 50 mM NaCl, 2 mM DTT with protease inhibitors) and solubilized by vortexing for 50 s. 100 µL buffer B (20 mM Tris-HCl, pH 8.0, 200 mM NaCl, 2 mM DTT) or 50 µL buffer B plus 50 µL 4% LMNG/CHS (8:1 mass ratio, solubilized in buffer B) were added into the solution mixture. Soluble IFT-A was separated by high-speed centrifugation (15,000 rpm for 45 min). The FSEC was performed with the running buffer containing the 20 mM Tris-HCl, pH 8.0, 200 mM NaCl, 2 mM DTT and 0.004% LMNG/CHS (8:1 mass ratio). All FSEC experiments were done on a Shimadzu HPLC system with an RF-20A fluorescence detection unit. The total area of the IFT-A peak was calculated by GraphPad Prism.

To investigate the role of zinc fingers in IFT-A complex stability, 10 mM TPEN (N,N,N’,N’-Tetrakis(2-pyridylmethyl)ethylenediamine) was added to the IFT-A protein (the peak fraction from size exclusion chromatography). Since the TPEN stock solution was prepared in ethanol, the same amount of ethanol was added in the IFT-A protein as a control group. Both samples were mixed and incubated on ice for 1 h followed by centrifugation (15,000 rpm, 30 min). The running buffer for FESC was composed of 20 mM Tris-HCl, pH 8.0, 200 mM NaCl, 2 mM DTT and 10 mM EDTA. The sample was autoloaded each hour and the amount of IFT-A complex was monitored by tryptophan fluorescence.

### Plasmids for imaging assay

pG-LAP1 (pCDNA5/FRT/TO-EGFP-TEV-Stag-X) was from Addgene.^72^ N-terminally LAP-tagged retroviral constructs of full length and truncations of TULP3 were generated by Gateway cloning into a gatewaytized LAP1 version of pBABE. Retroviral constructs of HA-INPP5E were generated by cloning into pQXIN vector. Single or multiple amino acid mutants of TULP3 were generated by Q5 site-directed mutagenesis (New England Biolabs).

### Cell culture and generation of stable cell lines

IMCD3 Flp-In and Phoenix A (PhA, Indiana University National Gene Vector Biorepository) cells were cultured in DMEM high glucose (Sigma-Aldrich; supplemented with 10% cosmic serum, 0.05 mg/mL penicillin, 0.05 mg/mL streptomycin, and 4.5 mM glutamine). Stable cell lines were generated by retroviral infection. WT and *Ift140* KO MEFs were gifts from Greg Pazour.^73^

### Generation of *Tulp3* KO cell lines

CRISPR/Cas9 KO lines for *Tulp3* were generated in IMCD3 Flp-In (Invitrogen) cells by cloning targeting sequences in exon 3 of *Tulp3* (ACGTCGCTGCGAGGCATCTG and TGGCTTTAACCTTCGCAGCC) into pLentiCRISPR backbone. Single clones were isolated using a serial dilution method. Clonal lines were tested for the KO by Sanger sequencing and immunoblotting for TULP3.^22^

### Immunofluorescence of cultured cells and microscopy

Cells were cultured on coverslips until confluency and starved for the indicated periods. Cells were fixed with 4% PFA. After blocking with 5% normal donkey serum, the cells were incubated with primary antibody solutions for 1 h at room temperature and treated with secondary antibodies for 30 min along with Hoechst 33342 (Invitrogen). Primary antibodies used were against the following antigens: acetylated tubulin (T6793; Sigma), γ-tubulin (GTU-88, Sigma; 1:500), ARL13B (gift of rabbit antiserum from Tamara Caspary) and GPR161 (affinity-purified rabbit polyclonal^74^). The coverslips were mounted using Fluoromount G (SouthernBiotech). Images were acquired on a widefield microscope (AxioImager.Z1; ZEISS) or a spinning disk confocal microscope (Nikon CSU-W1 SoRa). Images in the widefield microscope were acquired using a Plan Apochromat objective (40×/1.3 NA oil) and sCMOS camera (PCO Edge; BioVision Technologies) controlled using Micro-Manager software (University of California, San Francisco) at room temperature. Images in the spinning disk confocal microscope were acquired using a Plan Apochromat objective (100×/1.45 NA oil), an sCMOS camera (Hamamatsu Orca-Fusion), and a Piezo z-drive for fast z-stack acquisition controlled using Nikon NIS-Elements software at room temperature. Between 10 and 30 z sections at 0.2–0.8-µm intervals were acquired. For quantitative analysis of ciliary localization, stacks of images were acquired from 3–8 consecutive fields with confluent cells by looking into the DAPI channel, and percentages of protein-positive ciliated cells were counted. Maximal projections from images of stacks were exported from ImageJ/Fiji (National Institutes of Health) using similar parameters (image intensity and contrast) for image files from the same experiment. For measuring ciliary pixel intensities, image stacks were acquired with z sections at 0.8-µm intervals. An image interval with maximal intensity was chosen, and cilia were demarcated with a region of interest using the fluorescence signal for acetylated α-tubulin. The mean pixel intensities for the corresponding proteins were exported from ImageJ/Fiji.

### Immunoblotting

Cell pellets were lysed by resuspending and nutating for 20 min in 50 mM HEPES, pH 7.5, 150 mM NaCl, 1 mM EDTA, 0.1% Triton X-100, 1 mM AEBSF, and 0.01 mg/mL each of leupeptin, pepstatin, and chymostatin. Lysates were centrifuged at 12,000× *g* for 15 min and immunoblotted with antibody against S tag (mouse monoclonal MAC112), followed by visualization using IRDye-tagged secondary antibody and hFAB Rhodamine Anti-Tubulin (Bio-Rad; 12004166). Images were taken in a BioRad Chemidoc MP imaging system.

### Statistical analyses

Statistical analyses were performed using GraphPad Prism. Student’s *t*-test was used to compare two groups. For ciliary intensities, nonparametric Kruskal-Wallis one-way ANOVA and Dunn’s test for multiple comparisons between all possible pairs was used. Tukey’s multiple comparison test was performed to compare ciliary lengths between all possible pairs.

## Supplementary information

Supplementary information, Figure S1

Supplementary information, Figure S2

Supplementary information, Figure S3

Supplementary information, Figure S4

Supplementary information, Figure S5

Supplementary information, Figure S6

Supplementary information, Figure S7

Supplementary information, Figure S8

Supplementary information, Figure S9

Supplementary information, Figure S10

Supplementary information, Table S1

## Data Availability

Coordinates and related data for structures of IFT-A or IFT-A–TULP3 complexes have been deposited in the PDB and Electron Microscopy Data Bank (EMDB), respectively, with PDB code 8FGW and EMDB code EMD-29073 for IFT-A and PDB code 8FH3 and EMDB code EMD-29078 for the IFT-A–TULP3 complex.
