# Supplementary material for: Human IFT-A complex structures provide molecular insights into ciliary transport
Source: Cell Res. 2023 Feb 13;33(4):288–98. doi: 10.1038/s41422-023-00778-3 (PMC10066299; doi:10.1038/s41422-023-00778-3)
Supplement: Supplementary file 1 — Supplementary information, Figure S1 [file 41422_2023_778_MOESM1_ESM.pdf]

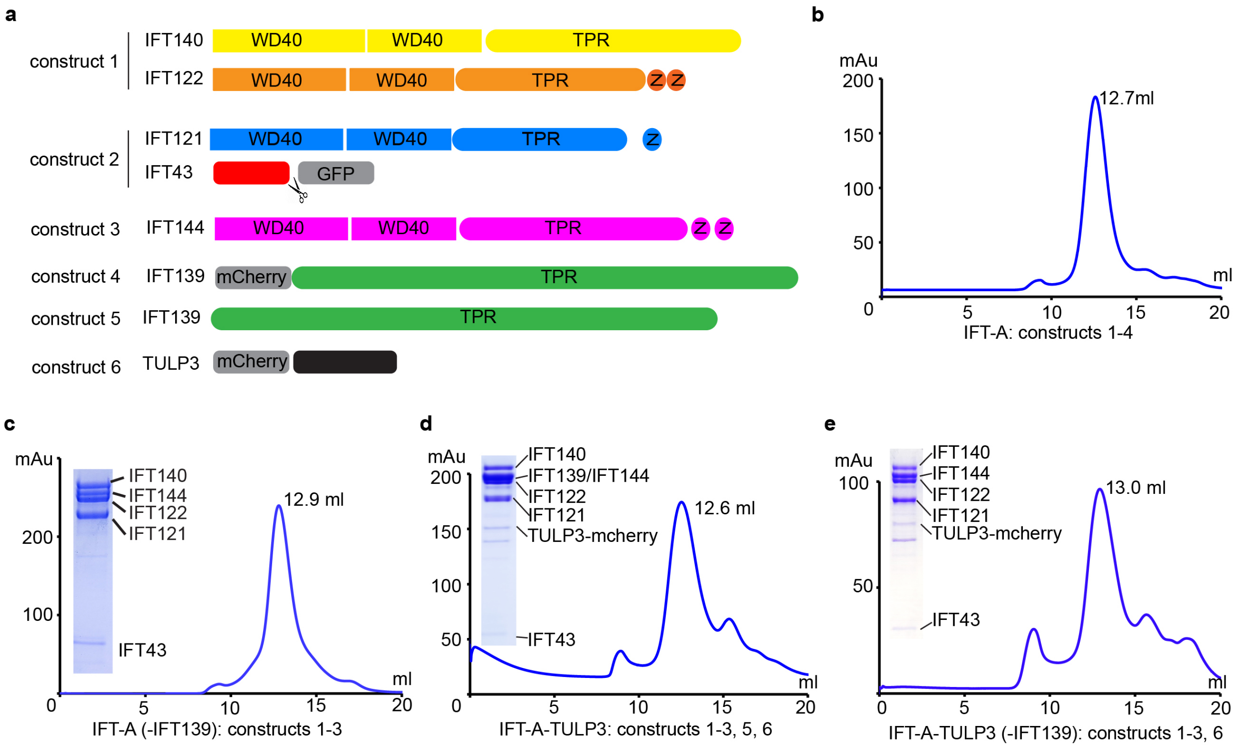

Supplementary information, Fig. S1: Purification of IFT-A and IFT-A–TULP3 complexes.

a, Constructs used for IFT-A expression and purification. Construct 1 contains IFT140 and IFT122, construct 2 contains IFT121 and IFT43, and constructs 3, 4, 5 and 6 express IFT144, mCherry-IFT139, IFT139 and mCherry-TULP3, respectively. IFT43 and IFT139 are tagged with eGFP and mCherry, respectively. The scissors icon indicates preScission protease site. **b**, The size exclusion chromatography of IFT-A complexes on a Superose 6 Increase column. **c-e**, The size exclusion chromatographies of IFT-A (no IFT139), IFT-A–TULP3 and IFT-A–TULP3 (no IFT139) complexes and the corresponding SDS-PAGEs of the peak fraction.
