# Supplementary material for: Human IFT-A complex structures provide molecular insights into ciliary transport
Source: Cell Res. 2023 Feb 13;33(4):288–98. doi: 10.1038/s41422-023-00778-3 (PMC10066299; doi:10.1038/s41422-023-00778-3)
Supplement: Supplementary file 2 — Supplementary information, Figure S2 [file 41422_2023_778_MOESM2_ESM.pdf]

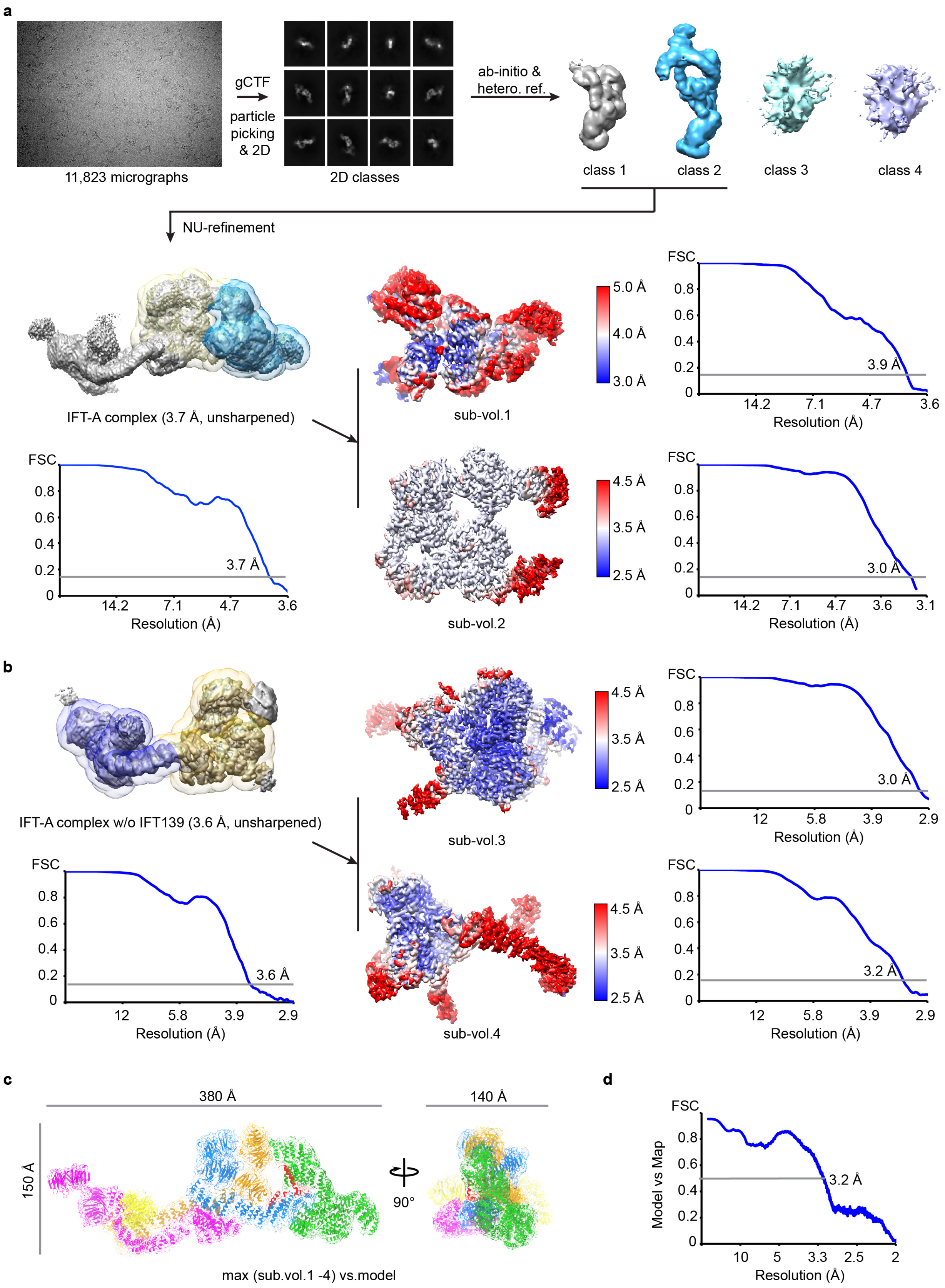
a
11,823 micrographs
gCTF
particle picking & 2D
2D classes
ab-initio & hetero. ref.
class 1
class 2
class 3
class 4
NU-refinement
IFT-A complex (3.7 Å, unsharpened)
sub-vol.1
5.0 Å
4.0 Å
3.0 Å
FSC
3.9 Å
Resolution (Å)
3.7 Å
sub-vol.2
4.5 Å
3.5 Å
2.5 Å
3.0 Å
b
IFT-A complex w/o IFT139 (3.6 Å, unsharpened)
sub-vol.3
3.0 Å
3.6 Å
sub-vol.4
3.2 Å
c
380 Å
140 Å
150 Å
90°
max (sub.vol.1 -4) vs.model
d
Model vs Map
3.2 Å

Supplementary information, Fig. S2: Structural determination of the IFT-A complex.

a, A simplified flowchart of the data process protocol of the IFT-A complex. Focused refinement was performed for two sub-volumes. Sub-volume 1 contains mainly IFT139 and IFT43, and sub-volume 2 contains most of the IFT122 and IFT121 chains. Local resolutions are calculated and shown. **b**, A simplified flowchart of the data process protocol of the IFT-A complex without IFT139. Focused refinement was performed for two sub-volumes. Sub-volume 3 contains mainly most of the IFT122 and IFT121 chains and sub-volume 4 contains most of the IFT144, IFT140 and part of the IFT122 chains. Local resolutions are calculated and shown. **c**, Composed IFT-A map with atomic models docked in. A composed map of the IFT-A complex was calculated in UCSF Chimera by combining sub-volumes 1-4 after aligning with the 3.7-Å full complex map in **a**. The dimensions of the complex are shown. **d**, the FSC curve between the IFT-A model and composite map.
