# Supplementary material for: Human IFT-A complex structures provide molecular insights into ciliary transport
Source: Cell Res. 2023 Feb 13;33(4):288–98. doi: 10.1038/s41422-023-00778-3 (PMC10066299; doi:10.1038/s41422-023-00778-3)
Supplement: Supplementary file 3 — Supplementary information, Figure S3 [file 41422_2023_778_MOESM3_ESM.pdf]

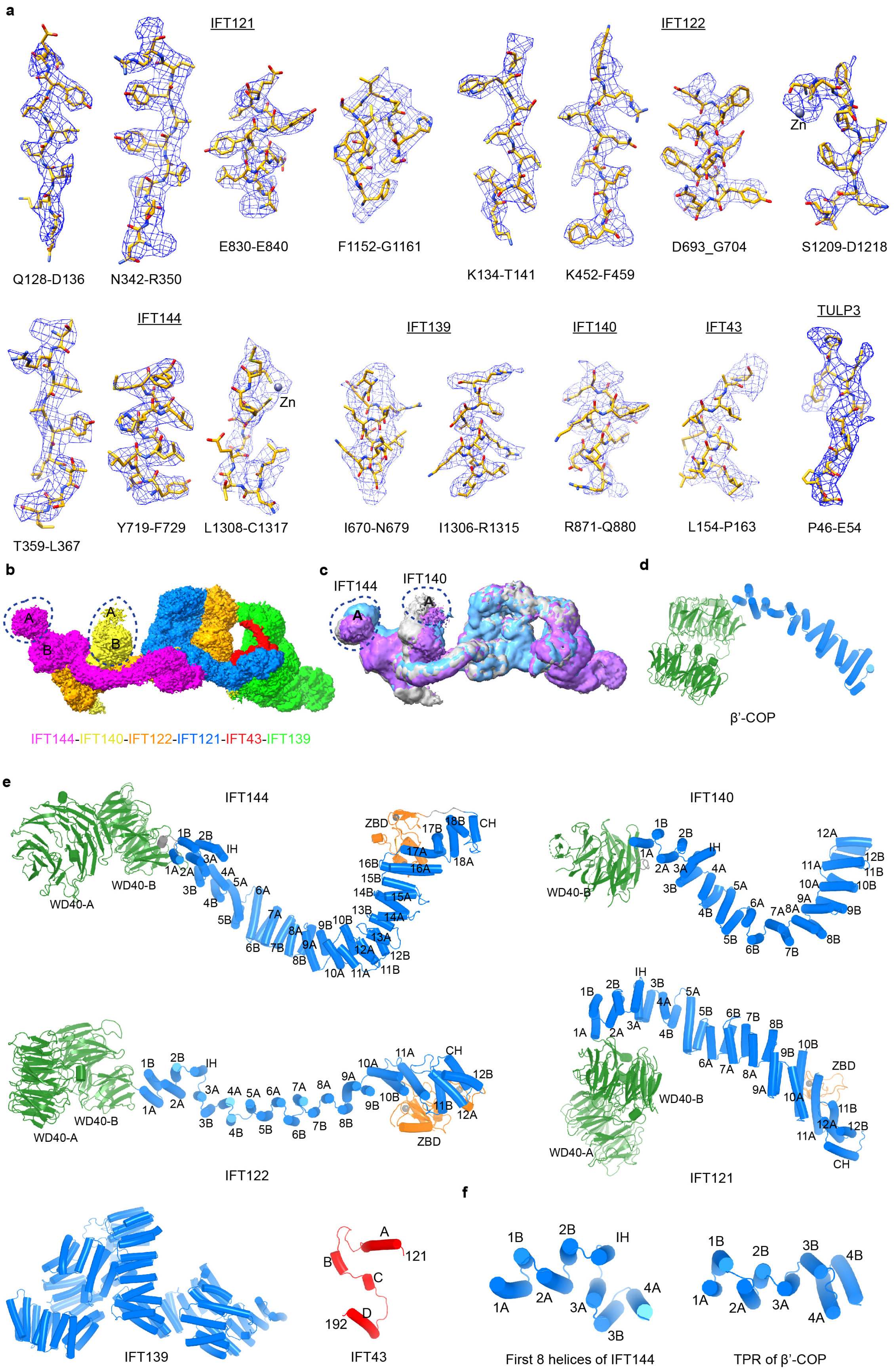
a
IFT121
IFT122
Q128-D136
N342-R350
E830-E840
F1152-G1161
K134-T141
K452-F459
D693_G704
S1209-D1218
Zn
IFT144
IFT139
IFT140
IFT43
TULP3
T359-L367
Y719-F729
L1308-C1317
I670-N679
I1306-R1315
R871-Q880
L154-P163
P46-E54
b
c
d
IFT144
IFT140
β'-COP
IFT144-IFT140-IFT122-IFT121-IFT43-IFT139
e
IFT144
IFT140
WD40-A
WD40-B
ZBD
CH
IH
IFT122
IFT121
f
IFT139
IFT43
121
192
First 8 helices of IFT144
TPR of β'-COP

Supplementary information, Fig. S3: Features of IFT-A complex subunits.

a, Representative local densities with refined model for each IFT-A subunit, zinc and TULP3. **b**, IFT-A map contoured at a low threshold. The flexible WD40 domains of IFT144 and IFT140 are indicated by dashed lines. **c**, Different conformations of IFT-A resulted from 3D classification. The flexible WD40-A domains of IFT140 and IFT144 are indicated by dashed circles. **d,** The structure of β'-COP. **e**, Structural model of each IFT-A subunit. WD40, TPR-like and zinc-binding domains are colored in green, blue and orange, respectively. **f**, The first 4 TPR or TPR-like repeats of IFT144 and β'-COP. The insertion helix of the IFT144 TPR-like domain is indicated as "IH".
