# Supplementary material for: Human IFT-A complex structures provide molecular insights into ciliary transport
Source: Cell Res. 2023 Feb 13;33(4):288–98. doi: 10.1038/s41422-023-00778-3 (PMC10066299; doi:10.1038/s41422-023-00778-3)
Supplement: Supplementary file 4 — Supplementary information, Figure S4 [file 41422_2023_778_MOESM4_ESM.pdf]

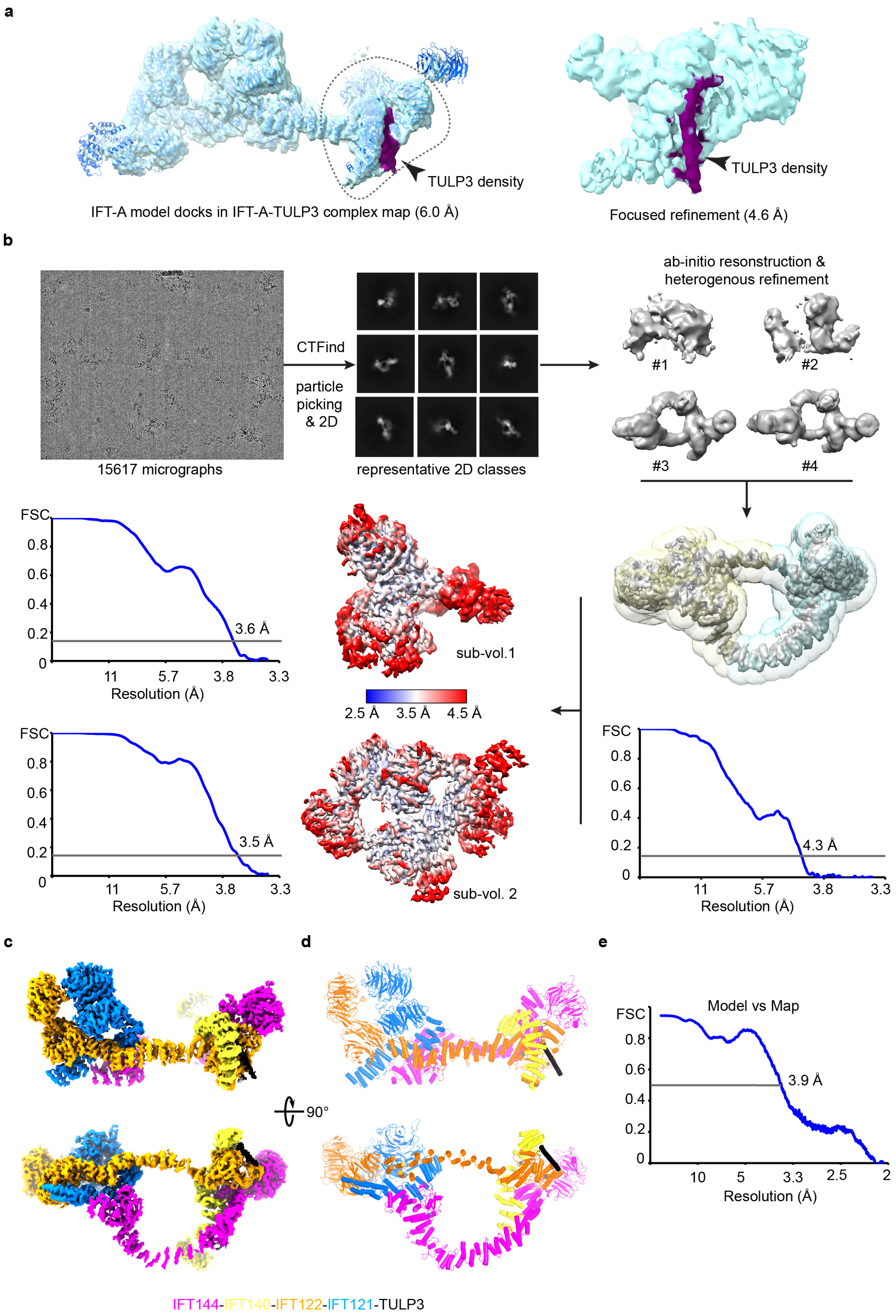
a
TULP3 density
IFT-A model docks in IFT-A-TULP3 complex map (6.0 Å)
TULP3 density
Focused refinement (4.6 Å)
b
15617 micrographs
CTFind
particle picking & 2D
representative 2D classes
ab-initio resonstruction & heterogenous refinement
#1
#2
#3
#4
FSC
0.8
0.6
0.4
0.2
0
3.6 Å
11
5.7
3.8
3.3
Resolution (Å)
sub-vol.1
2.5 Å
3.5 Å
4.5 Å
3.5 Å
sub-vol. 2
4.3 Å
c
d
e
90°
Model vs Map
3.9 Å
10
5
3.3
2.5
2
IFT144-IFT140-IFT122-IFT121-TULP3

Supplementary information, Fig. S4: Structural determination of the IFT-A–TULP3 complex.

a, Low-resolution map of the IFT-A–TULP3 complex**.** IFT-A and TULP3 are colored in cyan and purple, respectively. **b**, A simplified flowchart of the data process protocol of the IFT-A–TULP3 complex without IFT139. Focused refinement was performed for two sub-volumes. Sub-volume 1 contains the TULP3, IFT144, IFT140 and part of the IFT122 subunits and sub-volume 2 contains most of the IFT121 and IFT122 subunits. Local resolutions are calculated and shown. **c-d**, The composite map and atomic model of the IFT-A–TULP3 complex without IFT139. IFT43 is not resolved. **e,** the FSC curve between the model (**d**) and composite map (**c**).
