# Supplementary material for: Human IFT-A complex structures provide molecular insights into ciliary transport
Source: Cell Res. 2023 Feb 13;33(4):288–98. doi: 10.1038/s41422-023-00778-3 (PMC10066299; doi:10.1038/s41422-023-00778-3)
Supplement: Supplementary file 5 — Supplementary information, Figure S5 [file 41422_2023_778_MOESM5_ESM.pdf]

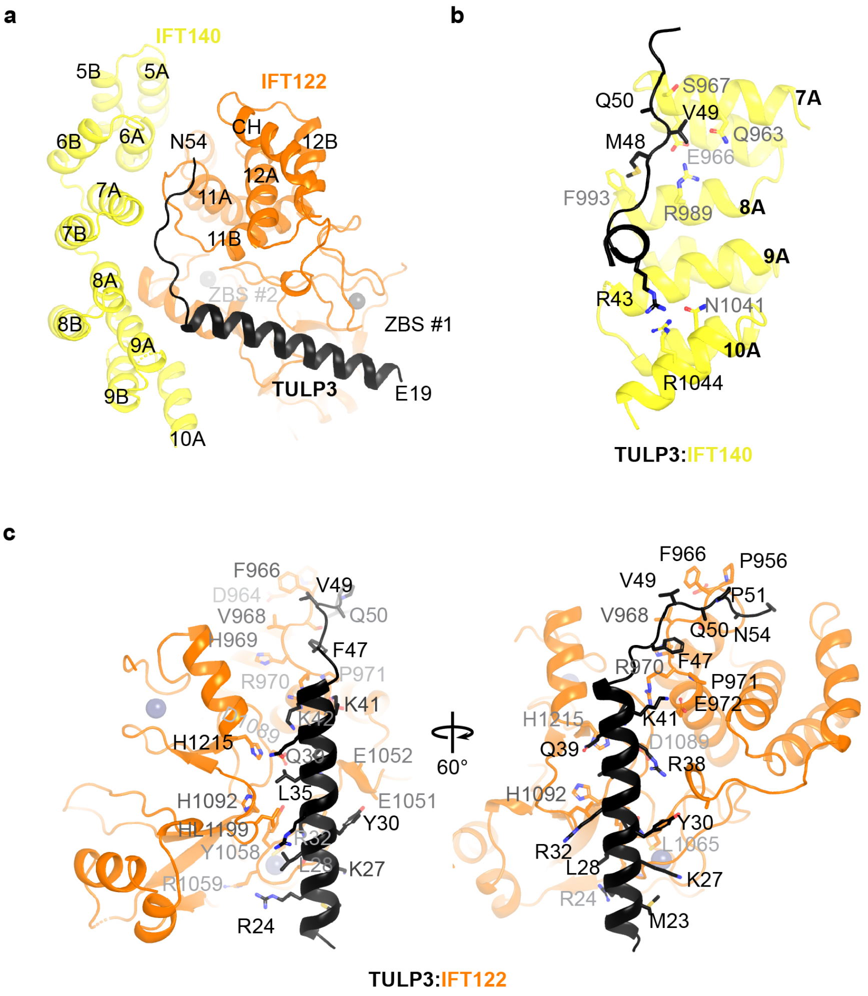


**Supplementary information, Fig. S5: Interactions between IFT-A and TULP3.**

**a,** The binding site of TULP3. **b,** Interactions between TULP3 and IFT140 with side chains of interacting residues shown as sticks. **c,** Interactions between TULP3 and IFT122.
