# Supplementary material for: Human IFT-A complex structures provide molecular insights into ciliary transport
Source: Cell Res. 2023 Feb 13;33(4):288–98. doi: 10.1038/s41422-023-00778-3 (PMC10066299; doi:10.1038/s41422-023-00778-3)
Supplement: Supplementary file 6 — Supplementary information, Figure S6 [file 41422_2023_778_MOESM6_ESM.pdf]

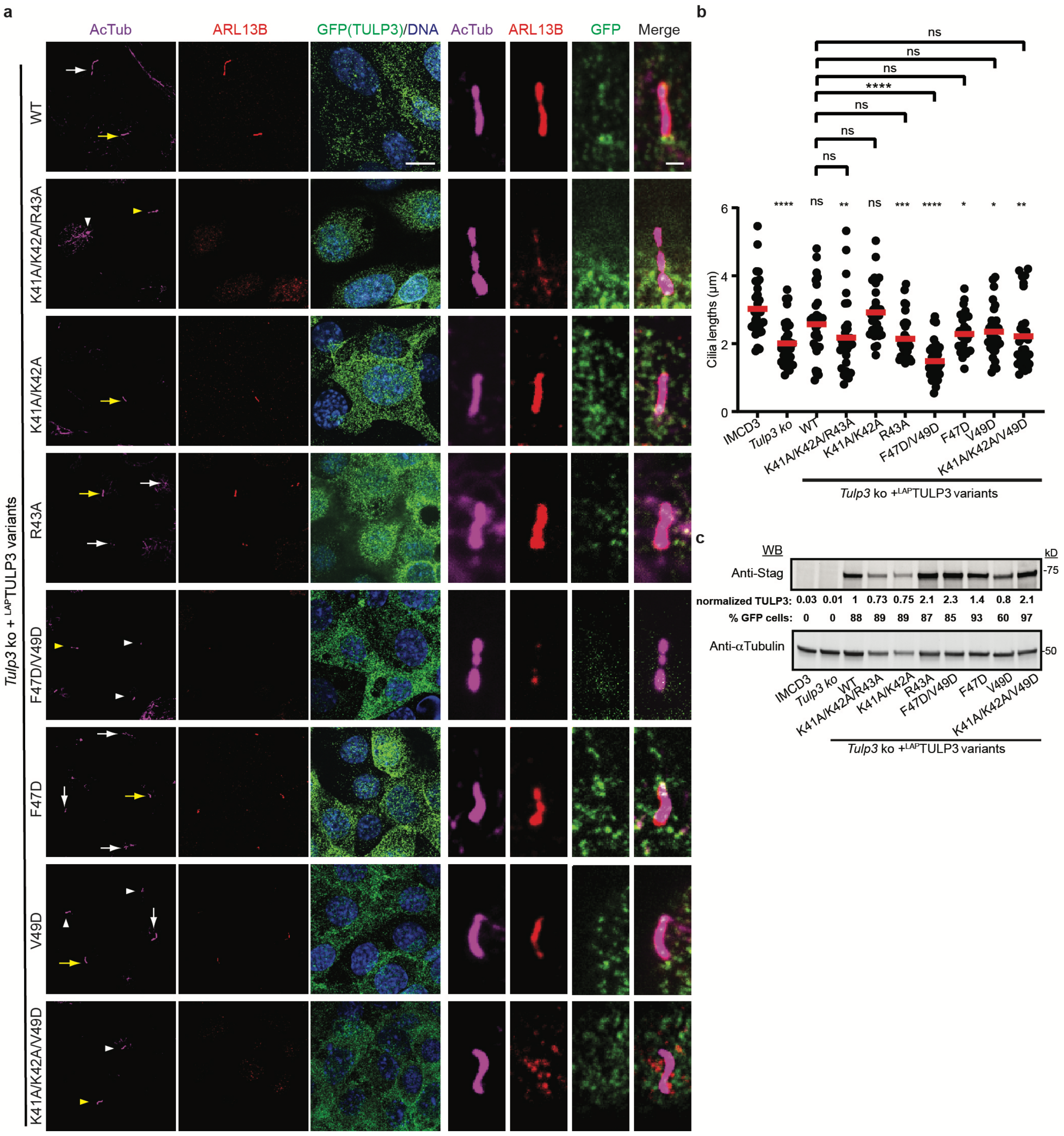

Supplementary information, Fig. S6: Ciliary trafficking of ARL13B with TULP3 mutants.

a, Representative cell images quantified in Fig. 4c are shown. *Tulp3* ko IMCD3 line stably expressing LAP-tagged wild type (WT) TULP3 or indicated mutant were grown to confluence, serum starved for 36 h before fixation and immunostained for ARL13B (red), GFP (green), acetylated tubulin (magenta) and counterstained for DNA. Maximum intensity Z-projections of

stacks at the plane of the cilia (red/magenta channels) or nuclei (green channel) are shown in the left panels. Arrows and arrowheads indicate cilia positive and negative for indicated proteins, respectively. Yellow arrow/arrowhead marked cilia are shown in the right insets that show maximum intensity Z-projection at the plane of the cilia. Scale, 5 μm; insets, 1 μm. **b,** Quantification of cilium length in the presence of wild type or LAP-tagged *TULP3* variants stably expressed in *Tulp3* ko IMCD3 cells. Comparisons of the lines with respect to IMCD3 or with respect to stably expressing LAP-tagged TULP3^{WT} in *Tulp3* ko cells are shown. ****, $p<0.0001$; ***, $p<0.001$; **, $p<0.01$; *, $p<0.05$.; ns, not significant **c,** Western blot of wild type or LAP-tagged *TULP3* variants stably expressed in *Tulp3* ko IMCD3 cells. The amounts of LAP-tagged TULP3 and mutants are normalized to α-tubulin. The percentage of GFP-expressing cells is also indicated. LAP, EGFP-TEV-S tag-X.
