# Supplementary material for: Human IFT-A complex structures provide molecular insights into ciliary transport
Source: Cell Res. 2023 Feb 13;33(4):288–98. doi: 10.1038/s41422-023-00778-3 (PMC10066299; doi:10.1038/s41422-023-00778-3)
Supplement: Supplementary file 7 — Supplementary information, Figure S7 [file 41422_2023_778_MOESM7_ESM.pdf]

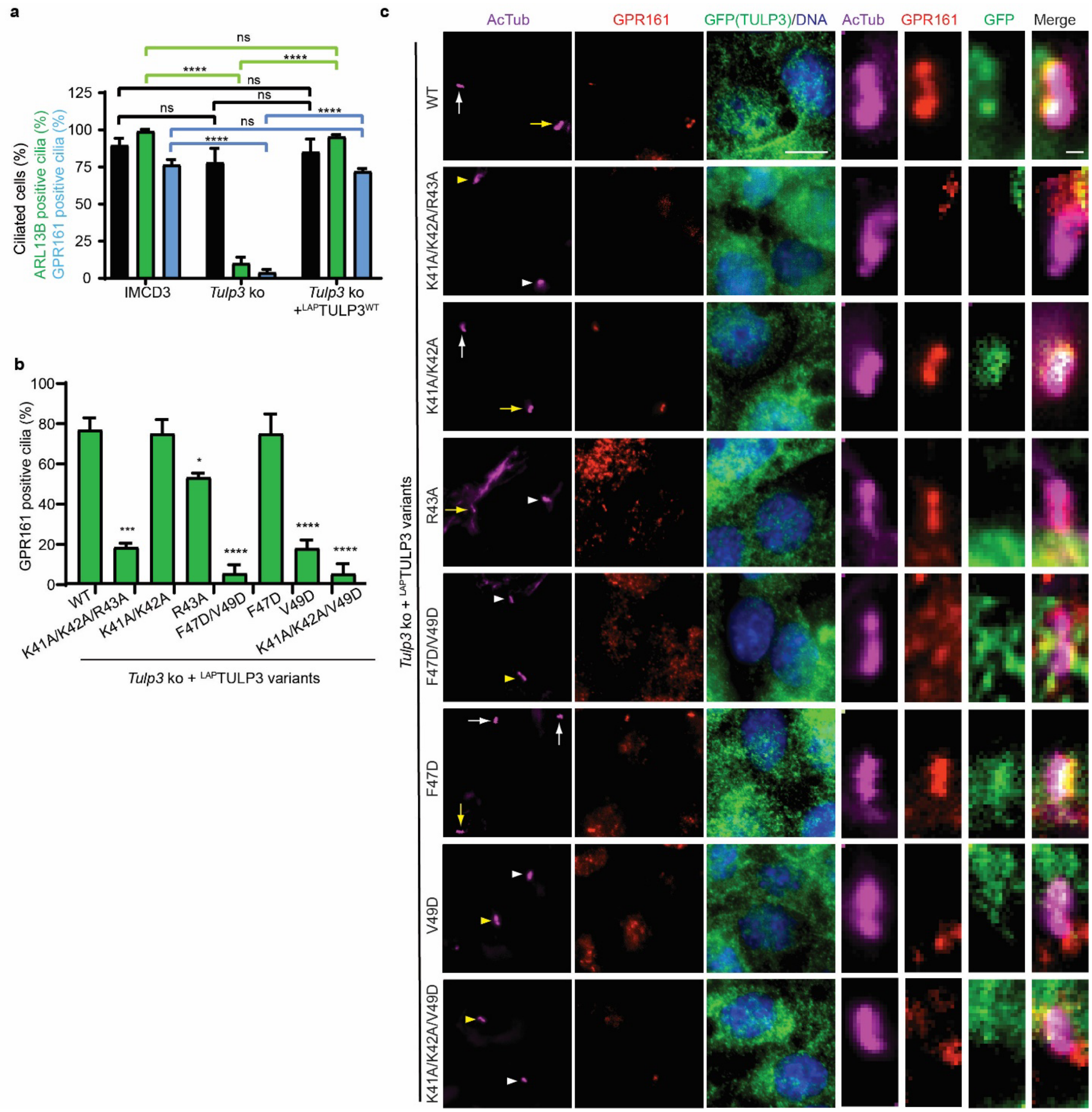

Supplementary information, Fig. S7: Ciliary trafficking of GPR161 in TULP3 mutants.

a, LAP-tag TULP3 rescues the ciliary transport of ARL13B and GPR161 in *Tulp3* ko IMCD3 cells without affecting ciliation percentage. ****, $p<0.0001$; ns, not significant. **b,** *Tulp3 ko* IMCD3 line stably expressing LAP-tagged wild type (WT) TULP3 or indicated mutants were grown to confluence, serum starved for 36 h before fixation and immunostained for GPR161, GFP, acetylated tubulin and counterstained for DNA. Cilia in GFP(TULP3) expressing cells counted

from 2 experiments, n>200/condition. Data represent mean ± SD. ****, $p<0.0001$; *, $p<0.05$. **c**, Representative images of cells quantified in panel **a** are shown. Cells were immunostained for GPR161 (red), GFP (green), acetylated tubulin (magenta) and counterstained for DNA. Maximum intensity Z-projections of stacks at the plane of the cilia (red/magenta channels) or nuclei (green channel) are shown in the left panels. Arrows and arrowheads indicate cilia positive and negative for indicated proteins, respectively. Yellow arrow/arrowhead marked cilia are shown in the right insets that show maximum intensity Z-projection at the plane of the cilia. Scale, 10 μm; insets, 1 μm.
