# Supplementary material for: Human IFT-A complex structures provide molecular insights into ciliary transport
Source: Cell Res. 2023 Feb 13;33(4):288–98. doi: 10.1038/s41422-023-00778-3 (PMC10066299; doi:10.1038/s41422-023-00778-3)
Supplement: Supplementary file 8 — Supplementary information, Figure S8 [file 41422_2023_778_MOESM8_ESM.pdf]

a

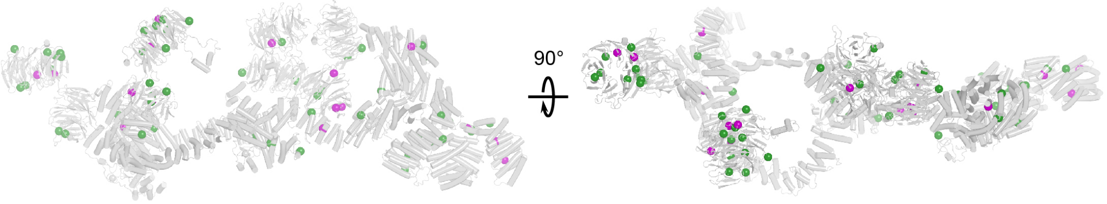

b

	IFT144	IFT140	IFT122	IFT121	IFT139	IFT43
exposed	L7, V68, G109, D159, F249, R272, N273, G294, D493, G495, R1178, D1235, C1267	H24, G140, L152, I233, E267, R280, Y311,T484, G522, R576, E664, E790, R871, S939	W7, S322, F570, G572, L712	W261, R478, E626, D841	P209, P466, S591, H566, D755, L795, R867, M1101, M1186, Y1167	W174
buried	A30, V345, L710	A212, V292, A418, C663, A974	V391, G495, V502	W331, A875	A44, W150, T231, A235, A499, L1202	n.a

Supplementary information, Fig. S8: Disease mutations of IFT-A complexes.

a, The disease mutations mapped based on solvent accessibility using the same model in Fig. 6. Green and purple spheres indicate exposed and buried residues, respectively. **b**, Solvent accessibility of disease mutations. The accessibility is calculated using chimera, and residues with <2.5 Å solvent accessible surface are considered buried.
