# Supplementary material for: Human IFT-A complex structures provide molecular insights into ciliary transport
Source: Cell Res. 2023 Feb 13;33(4):288–98. doi: 10.1038/s41422-023-00778-3 (PMC10066299; doi:10.1038/s41422-023-00778-3)
Supplement: Supplementary file 9 — Supplementary information, Figure S9 [file 41422_2023_778_MOESM9_ESM.pdf]

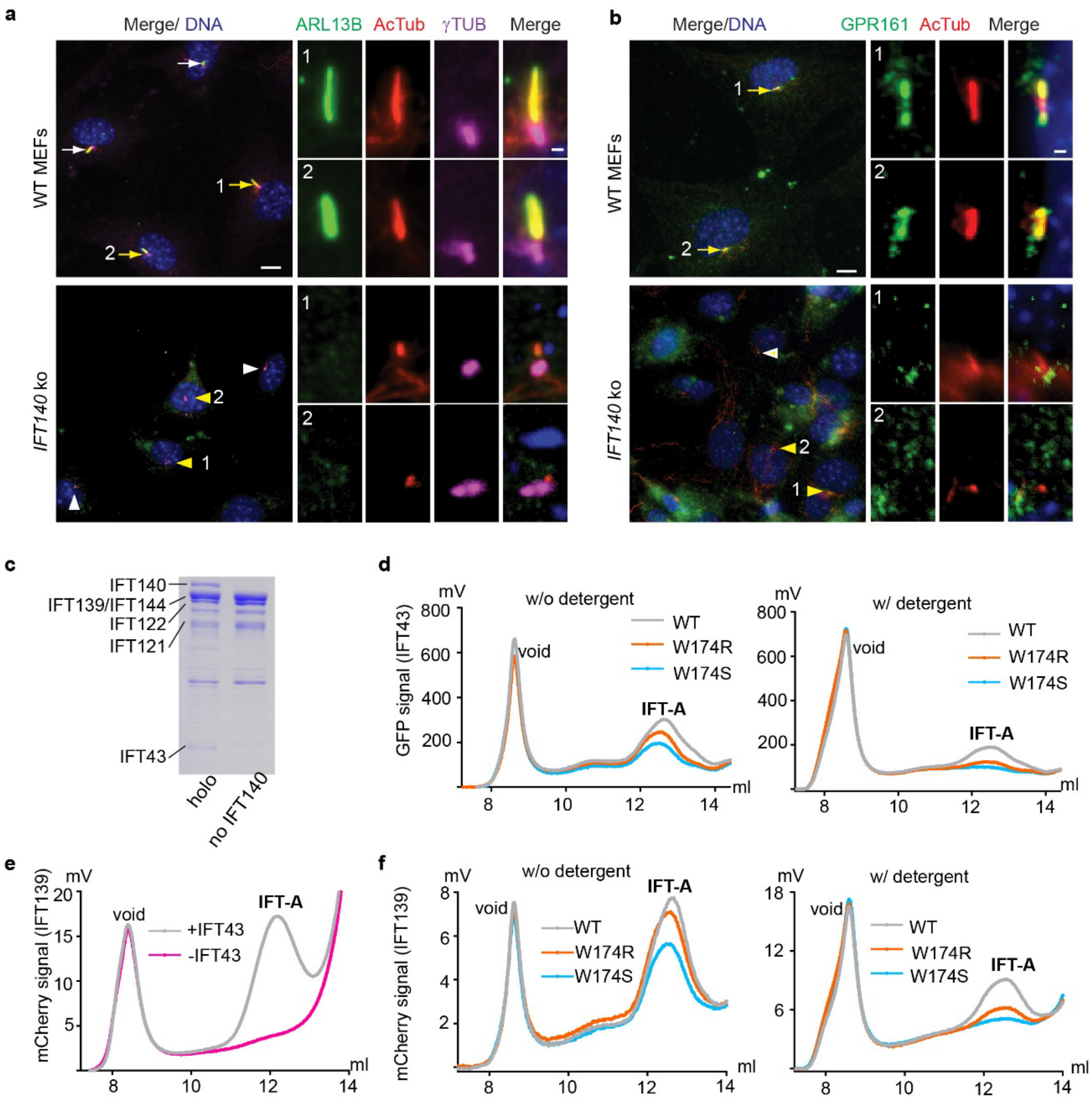

Supplementary information, Fig. S9: Disease mutations of IFT140 and IFT43.

a and b, Wild-type and *Ift140* ko MEFs were serum starved upon confluence for 48 h before fixation. Fixed cells were immunostained for ARL13B (green) or GPR161 (green), acetylated tubulin (AcTub, red), γ-tubulin (magenta) and counterstained for DNA (blue). Quantification shown in Fig 6c. Arrows indicate cilia positive for ARL13B or GPR161. Arrowheads indicate cilia lacking ARL13B or GPR161 or no cilia. Yellow arrow/arrowhead marked regions are shown in the insets. Scale bar, 10 μm; insets, 1 μm. **c**, The SDS-PAGE of purified IFT-A complex (with or without

IFT140). **d,** FSEC traces of IFT-A complex formation in the absence and presence of detergents and IFT43 mutations by monitoring the GFP fluorescence of GFP-IFT43. **e,** FSEC traces of IFT-A complex formation with or without GFP-IFT43 by monitoring the mCherry fluorescence of IFT139 in the presence of detergents. The rising peak after 14 ml due to the excess of mCherry-IFT139 protein alone or mCherry-IFT139-containing subcomplexes is not shown for clarity. **f,** FSEC traces of IFT-A complex formation monitored by the mCherry fluorescence of IFT139 in the presence and absence of IFT43 mutations with or without detergent stress.
