# Supplementary material for: Human IFT-A complex structures provide molecular insights into ciliary transport
Source: Cell Res. 2023 Feb 13;33(4):288–98. doi: 10.1038/s41422-023-00778-3 (PMC10066299; doi:10.1038/s41422-023-00778-3)
Supplement: Supplementary file 10 — Supplementary information, Figure S10 [file 41422_2023_778_MOESM10_ESM.pdf]

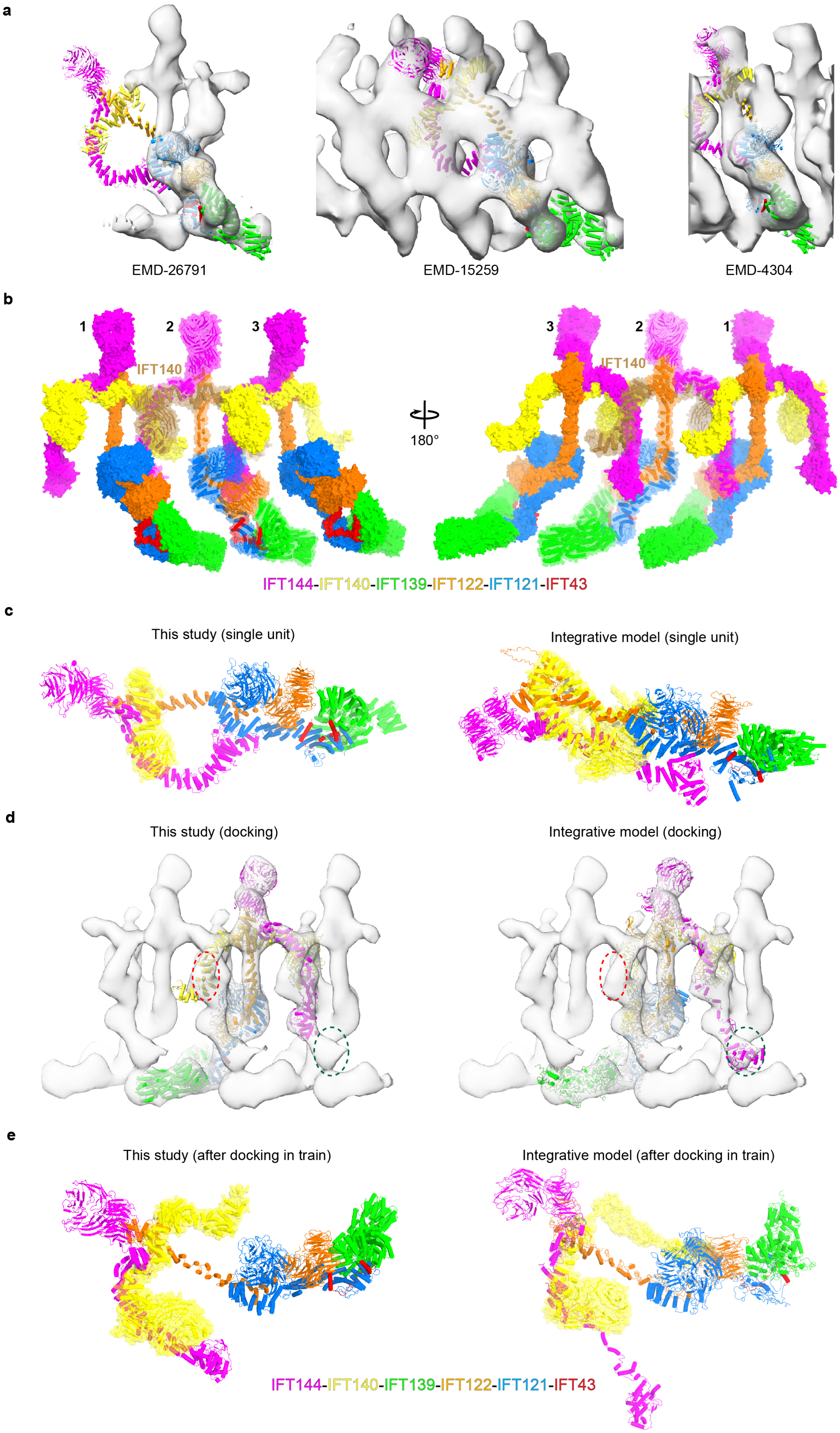

a
EMD-26791
EMD-15259
EMD-4304
b
1
2
3
IFT140
180°
3
2
1
IFT140
IFT144-IFT140-IFT139-IFT122-IFT121-IFT43
c
This study (single unit)
Integrative model (single unit)
d
This study (docking)
Integrative model (docking)
e
This study (after docking in train)
Integrative model (after docking in train)
IFT144-IFT140-IFT139-IFT122-IFT121-IFT43

Supplementary information, Fig. S10: Model of the IFT-A train.

a, Rigid-body-based docking of the IFT-A model to the cryo-ET maps (EMD-26791, EMD-15259 and EMD-4304), showing non-perfect fitting. **b**, Model of IFT-A polymer with three copies of IFT-A. IFT140 of the middle copy is colored in wheat for clarity. **c**, The conformation differences between our IFT-A structure and the integrative model as monomers. **d**, The differences between the docking results of our work and the integrative model. Red dashed circles indicate the C-terminal part of IFT140, and green dashed circles indicate unexplained density in our study. **e,** The differences in the IFT-A models after docking into the cryo-ET map.
