# Supplementary material for: Human IFT-A complex structures provide molecular insights into ciliary transport
Source: Cell Res. 2023 Feb 13;33(4):288–98. doi: 10.1038/s41422-023-00778-3 (PMC10066299; doi:10.1038/s41422-023-00778-3)
Supplement: Supplementary file 11 — Supplementary information, Table S1 [file 41422_2023_778_MOESM11_ESM.pdf]

Supplementary information, Table S1. Cryo-EM data collection, refinement and validation statistics

	IFT-A	IFT-A (-IFT139)	IFT-A–TULP3 (-IFT139)	IFT-A–TULP3
	(EMDB: EMD-29073) (PDB: 8FGW)		(EMDB: EMD-29078) (PDB: 8FH3)	
**Data collection and processing**				
Magnification	81,000	105,000	81,000	63,000
Voltage (kV)	300	300	300	200
Electron exposure (e–/Å^2)	66.2	76.2	53.06	82.5
Defocus range (μm)	-0.6 – -1.8	-0.6 – -1.8	-0.6 – -2.0	-0.6 – -1.8
Pixel size (Å)	0.5285	0.413	0.5285	0.6572
Symmetry imposed	C1	C1	C1	C1
Initial particle images (no.)	1,649,804	1,962,671	2,136,547	485,077
Final particle images (no.)	67,560	180,200	113,784	61,090
Map resolution (Å)	Overall: 3.7<br>Sub-vol.1: 3.9<br>Sub-vol.2: 3.0	Overall: 3.6<br>Sub-vol.3: 3.0<br>Sub-vol.4: 3.2	Overall: 4.3<br>Sub-vol.1: 3.6<br>Sub-vol.2: 3.5	6.0
FSC threshold	0.143	0.143	0.143	0.143
Map resolution range (Å)				
**Refinement**				
Initial model used (PDB code)	*De novo*/AF2		*De novo*/AF2	
Model resolution (Å)	3.2		3.9	
FSC threshold	0.5		0.5	
Model resolution range (Å)	3.2 – 57.5		3.9 – 50	
Map sharpening *B* factor (Å^2)	Sub-vol.1: -97.0<br>Sub-vol.2: -72.6<br>Sub-vol.3: -88.2<br>Sub-vol.4: -77.5		Sub-vol.1: -68.7<br>Sub-vol.2: -89.2	
Model composition				
Non-hydrogen atoms	43596		26774	
Protein residues	5564		3642	
Ligands	5		5	
B factors (Å^2)				
Protein	69.318		62.157	
Ligand	75.272		189.004	
R.m.s. deviations				
Bond lengths (Å)	0.015		0.010	
Bond angles (°)	0.852		0.983	
Validation				
MolProbity score	2.02		2.17	
Clashscore	13.25		15.79	
Poor rotamers (%)	0		0	
Ramachandran plot				
Favored (%)	94.26		92.57	
Allowed (%)	5.74		7.43	
Disallowed (%)	0		0	
